# Peptides for Vaccine Development

**DOI:** 10.1021/acsabm.1c01238

**Published:** 2022-02-23

**Authors:** Ian W. Hamley

**Affiliations:** Department of Chemistry, University of Reading, Whiteknights, Reading RG6 6AD, U.K.

**Keywords:** Peptides, vaccines, immune response, infectious diseases, cancer, epitopes, adjuvants

## Abstract

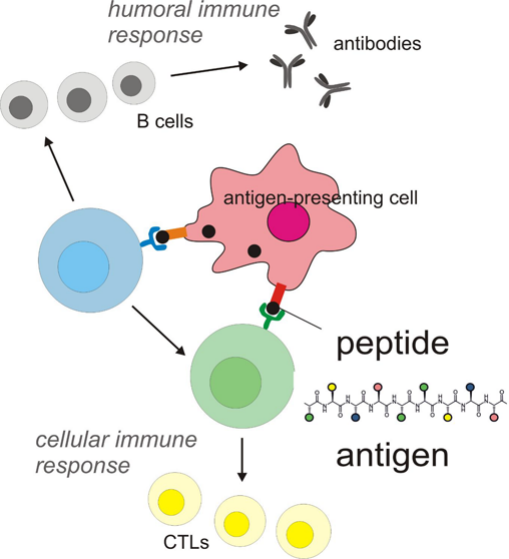

This review discusses peptide epitopes used as antigens in the development of vaccines in clinical trials as well as future vaccine candidates. It covers peptides used in potential immunotherapies for infectious diseases including SARS-CoV-2, influenza, hepatitis B and C, HIV, malaria, and others. In addition, peptides for cancer vaccines that target examples of overexpressed proteins are summarized, including human epidermal growth factor receptor 2 (HER-2), mucin 1 (MUC1), folate receptor, and others. The uses of peptides to target cancers caused by infective agents, for example, cervical cancer caused by human papilloma virus (HPV), are also discussed. This review also provides an overview of model peptide epitopes used to stimulate non-specific immune responses, and of self-adjuvanting peptides, as well as the influence of other adjuvants on peptide formulations. As highlighted in this review, several peptide immunotherapies are in advanced clinical trials as vaccines, and there is great potential for future therapies due the specificity of the response that can be achieved using peptide epitopes.

## 1 Introduction

The development of vaccines is of immense interest in view of existing and emerging viral diseases. Vaccination as currently recognized was developed and widely implemented starting just over 200 years ago, but variolation using cowpox to treat smallpox as used in China and Africa predates this by centuries. Many vaccines are based on inactivated pathogens; however, there is intense interest into methods based on modern biotechnologies, for example, application of DNA/RNA technologies, use of recombinant proteins, and virus-like nanoparticle formation. These have led recently, for instance, to vaccines for COVID-19, brought into practice remarkably rapidly to the huge benefit of humanity, saving hundreds of thousands of lives.^1−6^ Biotechnologies can provide a more targeted immune response, by biomolecular design and engineering, and in addition these techniques can be used to rapidly re-engineer vaccines in response to emerging variants and mutants. These characterize many diseases caused by coronaviruses, influenza virus, and others.

**Figure 1:**
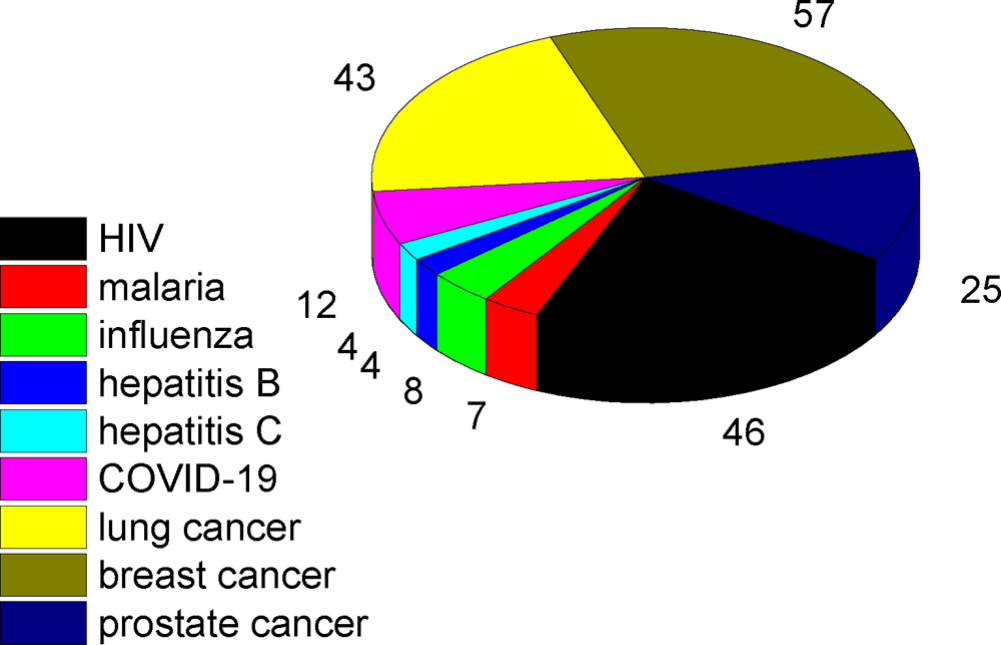
Number of clinical trials in progress for selected conditions (from www.clinicaltrials.gov, Oct 5, 2021, excluding withdrawn and terminated studies).

Subunit vaccines are attracting considerable attention due to the potential to precisely tune the immune response using antigens from protein fragments or peptides, as well as the relative ease of production of these biomolecules. In addition, peptides have potential activities as adjuvants. Short peptides can be produced at scale using automated synthesis methods, whereas longer peptides and proteins may conveniently be produced recombinantly. Certain types of peptides including surfactant-like peptides, lipopeptides (peptide amphiphiles), and amyloid-forming peptides can self-assemble forming nanostructures (nanofibrils, micelles, etc.) in aqueous solutions.^7−13^ This can be beneficial to the immunogenicity due to the high density presentation of bioactive peptide units, leading potentially to improved antigen or adjuvant efficacy. Peptides can form self-assembled peptide nanoparticles (SAPNs), and protein sub-units can assemble into virus-like particles (VLPs). Reviews on the use of such structures for vaccine development are available.^14−18^ The latter topic, since it concerns protein superstructures (recently reviewed elsewhere^19^) is outside the focus of the present review. As yet, few peptide-based vaccines have been employed in the clinic, although several systems are in advanced stages of clinical trials or are currently in active development (see Table 1 for examples).^20−25^ Examples of these studies are discussed in the current review. Figure 1 shows a representation of the approximate numbers of peptide vaccines under development for the same selection of conditions in Table 1. This is illustrative that most peptide vaccines are in development for cancers, with a significant fraction for HIV and smaller numbers for infectious viral diseases, with the exception of COVID-19 where many trials have recently been launched due to the recent impact of the global pandemic. The relatively smaller numbers of trials for other infectious diseases may reflect a number of factors including the prevalence of existing non-peptide vaccines (e.g., those in use based on inactivated viruses) for many viral diseases and the focus of pharmaceutical and academic researchers on conditions that affect affluent societies.

**Table 1:** Examples of Peptide-Based Candidate Vaccines in Active or Completed Phases of Development^a^

name	condition	composition	responsible/refs	phase, date of update
Multimeric-001 (M-001)	influenza	influenza hemagglutinin peptides (seeTable 3) along with standard (inactivated virus) vaccine	NIAID, USA^26−28^	II, Jun 18, 2020
BIPCV/IMX (V512)	influenza	influenza viral peptides	Merck, Sharp and Dohme	I, Feb 12, 2015
HCV antigen vaccine	hepatitis C	HCV antigen peptide	Valneva Austria GmbH	II, Oct 19, 2012
Pevion Biotech’s HCV vaccine candidate	hepatitis C	peptide CTL and Th epitopes in virosome-based formulation	CHUV Lausanne, Switzerland	I, Feb 8, 2010
FP-02.2 Vaccine	hepatitis B	nine HBV T cell epitope peptides	Altimmune, Inc., USA	I, Jan 9, 2019
HIV vaccine	HIV	highly conserved HIV-1 derived peptides and influenza matrix peptide	University of Pittsburgh, USA	I, Aug 27, 2007
HIV-1 C4–V3 polyvalent peptide vaccine	HIV	peptide epitopes from four of the most common HIV isolates in the United States and Europe and Th and CTL epitopes	Duke University, USA,^29^ and NIAID and others^30^	I, May 6, 2013; I, May 18, 2012 (mixture with IL-12); I, May 14, 2012 (with specific adjuvant)
AFO-18	HIV	mixture of CD8 and CD4 T cell epitopes (HLA-A*0201 epitopes)	Department of Infectious Diseases, Hvidovre University Hospital Copenhagen, Denmark^31−33^	I, Mar 27, 2014
UBI Vac (HIV-1 MN branched octameric V3 peptide vaccine)	HIV	branched peptide containing gp120 V3 sequence	University of California at San Francisco, USA,^34^ and St Louis University USA with University of Rochester, USA^35^ and others in studies of different formulations and administrations^36^	I, Jun 24, 2005
HIV CTL MEP vaccine	HIV	HIV CTL multi-epitope peptide vaccine	Wyeth (now Pfizer)	I, Dec 5, 2007
Peptides (N, R&C) formulated in Montanide ISA 720 or 51	malaria	mixtures of N, R, and C LSP derived from the *P. vivax* CS protein	Malaria Vaccine and Drug Testing Center Cali, Colombia	I, Mar 8, 2020
P27A	malaria	unstructured 104mer synthetic peptide from a *P. falciparum* protein	CHUV CRC Lausanne, Switzerland^37^	I, Jul 18, 2018
EpiVac	COVID-19	peptide antigens of SARS-CoV-2 proteins conjugated to a carrier protein	Federal State Budgetary Institution of Healthcare, Novosibirsk, Russia^38^	III, Aug, 27 2021
pVac	COVID-19	multipeptide	University Hospital Tübingen, Germany	I, Sept 8, 2021
UB-612	COVID-19	S1-RBD-protein based vaccine incorporating a Th/CTL epitope pool of peptides that bind MHC-I and MHC-II to	China Medical University Hospital Taichung, Taiwan	I, June 7, 2021
vaccine based on antigenic peptides	cancer (melanoma)	Melan-A peptide, influenza matrix peptide, Mage-A10 peptide	Ludwig Institute for Cancer Research and Multidisciplinary Oncology Center at the Centre Hospitalier Universitaire Vaudois Lausanne, Switzerland^39^	I, Apr 24, 2013
multi-epitope peptide vaccine for melanoma	cancer (melanoma)	tyrosinase and gp100 peptides	Memorial Sloan-Kettering Cancer Center, New York, USA	I, Jun 10, 2011
melanoma vaccine with peptides and leuprolide	cancer (melanoma)	peptide epitopes from gp100 and MAGE-3 with and without a LHRH agonist-leuprolide	University of Texas, Houston, USA	II, Oct 16, 2019
Long Peptide Vaccine (LPV7)	cancer (melanoma)	mixture of seven long peptide epitopes from gp100, tyrosinase, NY-ESO-1, MAGE-A1, and MAGE-A10	University of Texas, Houston, USA^40^	II, Nov 17, 2020
breast cancer vaccine	cancer (breast cancer)	nine peptides from HER-2/neu, carcinoembryonic antigen and cancer testis antigen	University of Virginia, Charlottesville, USA	I, Dec 16, 2016
multipeptide vaccine for advanced breast cancer	cancer (breast cancer)	hTERT (express telomerase) peptide and CTL peptide epitopes selected for low-affinity binding to HLA-A*02	University of Pennsylvania, Philadelphia, USA	1, Sept 29, 2016
folate receptor alpha peptide vaccine for breast cancer	cancer (breast cancer)	folate receptor α peptide	Marker Therapeutics, Inc.	II, Jul 19, 2021
peptide mixture vaccine for prostate cancer	cancer (prostate cancer)	NY-ESO-1 peptide epitopes	Baylor College of Medicine Houston, USA^41^	I, Nov 6, 2012
TARP peptide vaccine for prostate cancer	cancer (prostate cancer)	epitope-enhanced TARP peptide	National Institutes of Health Clinical Center Bethesda, USA	I, Aug 13, 2021
prostate-specific antigen peptide vaccine for prostate cancer	cancer (prostate cancer)	PSA peptide vaccine	University of Maryland, Baltimore, USA^42^	II, Jan 23, 2013
MUC1 peptide vaccine for triple-negative breast cancer	cancer (breast cancer)	MUC1 peptide	Case University Cleveland, USA	Jul 23, 2018
MUC1 peptide vaccine for lung cancer	cancer (lung cancer)	MUC1 peptide vaccine	Vaxil therapeutics Ltd	II, Aug 9, 2013
UV1	cancer (lung cancer)	three long peptides containing multiple epitopes from previous hTERT vaccination trials	Oslo University Hospital, Oslo, Norway^43^	I/IIa, May 17, 2021
HER-2/neu peptide antigen for various cancers	cancer (lung, ovarian, and breast cancers)	HER-2/neu peptide antigen	University of Washington, Seattle, USA	I, Feb 27, 2019

a Information from www.clinicaltrials.gov. There are large numbers of peptide vaccine candidates for HIV and cancers, especially melanoma, breast, prostate, and lung cancer, and only a few examples are listed here. More complete lists are available at the website. Abbreviations: NIAID, National Institute of Allergy and Infectious Diseases; HCV, hepatitis C virus; HBV, hepatitis B virus; CTL, cytotoxic T-lymphocyte; Th, helper T cell; CHUV, Vaccine and Immunotherapy Center; HIV, human immunodeficiency virus; LSP, long synthetic peptide; COVID-19, coronavirus disease 2019; SARS-CoV-2, severe acute respiratory syndrome coronavirus 2; RBD, receptor binding domain; MHC, major histocompatibility complex; NY-ESO-1, cancer–testis antigen 1; MAGE, melanoma antigen-encoding gene; LHRH, luteinizing hormone-releasing hormone; hTERT, human telomerase reverse transcriptase; TARP, T cell receptor gamma-chain alternate reading frame protein; PSA, prostate-specific antigen; MUC1, mucin 1; HER-2/neu, human epidermal growth factor receptor 2.

Peptide-based immunotherapies are also of great interest as cancer treatments. Cancer immunotherapies include those based on T cell transfer including CAR (chimeric antigen receptor) T cell therapy, monoclonal antibodies, immune system modulators such as interferons and interleukins, immune checkpoint inhibitors, and potentially peptide subunit vaccines. Many of these approaches can benefit from peptides; for example, molecules based on TLR (Toll-like receptor) agonist peptides have attracted attention in cancer immunotherapies.^44,45^ Here, peptide epitope vaccines for cancer immunotherapies are reviewed, along with peptide vaccines for a range of infectious diseases.

**Figure 2:**
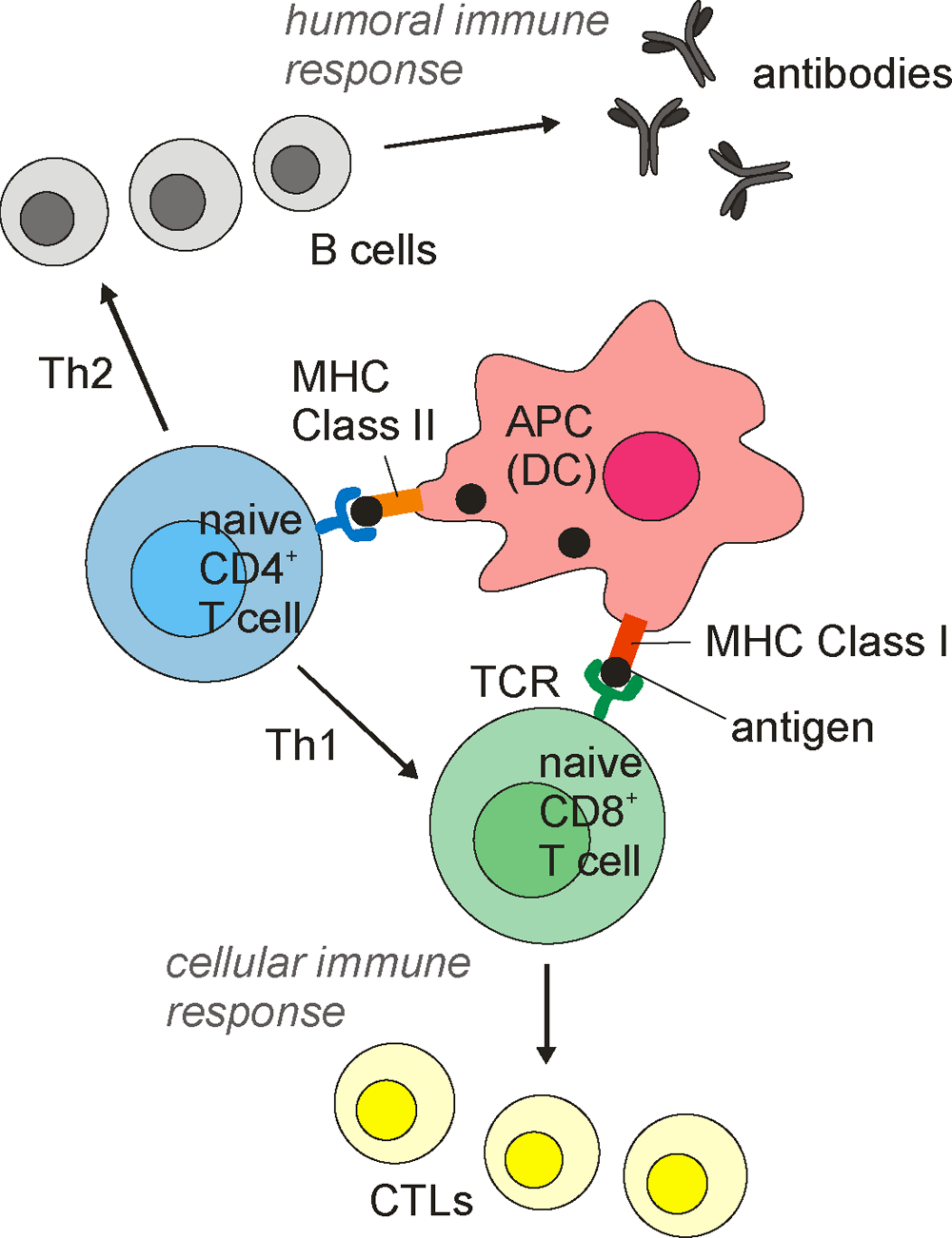
Antigen presentation and interaction with T-cells in the adaptive immunity system. Cell types and processes are discussed in the text. For simplicity, cell produced cytokines are not shown. Abbreviations: APC, antigen-presenting cell; DC, dendritic cell; MHC, major histocompatibility complex; TCR, T cell receptor; CTL, cytotoxic T-lymphocyte.

The immune system involves the innate and the adaptive systems. The former uses cells including neutrophils, macrophages, natural killer cells, and dendritic cells. The adaptive immune system relies on the activation of antigen-presenting cells (APCs) of the innate immune system. Antigen presentation involves the binding of antigen to the major histocompatibility complex (MHC), followed by transport of the complex to the cell surface where it can be recognized by a T cell receptor (TCR). This is illustrated in Figure 2. Two types of MHC interact with cytosolic intracellular peptides (MHC class I) or with peptides or proteins in endosomes or lyosomes after internalization (MHC class II molecules). The MHC-I/peptide complex activates naive (immature) CD8^+^ T cells to produce cytotoxic T cells (*T*_c_ or killer T-cells also known as cytotoxic T-lymphocytes (CTLs), a type of white blood cell) as shown in Figure 2 (CD = cluster of differentiation, cell surface glycoproteins that serve as ligands or receptors). Human leukocyte antigens (HLAs) present peptides on MHC-I after processing of antigen proteins in the proteasome, which are then destroyed by CTL cells. In contrast, in MHC class II, antigens are presented to CD4^+^ T cells. Proliferating helper T (Th) cells (a distinct kind of white blood cell) that produce effector T cells differentiate into Th1 and Th2 cell subtypes. Th1 helper cells generate a greater cell-mediated response mainly via CTLs and macrophages, with CD8^+^ T cells as effector cells (Figure 1). Th2 cells elicit a humoral immune response via CD4^+^ effector T cells, which activate, through a range of cytokines, B cells (produced from stem cells in bone marrow) and macrophages. CD4^+^ T cells also send signals via Th1 to CTLs. In turn, B cells produce (via cytokine stimulation) antibodies as part of the humoral immune response (Figure 2). B cells and macrophages, in addition to APCs such as dendritic cells (DCs), present MHC-II at high levels, and thus MHC-II molecules are expressed in a more cell-specific manner than those of MHC-I. Antigenic peptide binding by class I and class II MHCs has been reviewed.^46^ Databases of MHC ligands including peptides have been assembled.^47−53^

**Figure 3:**
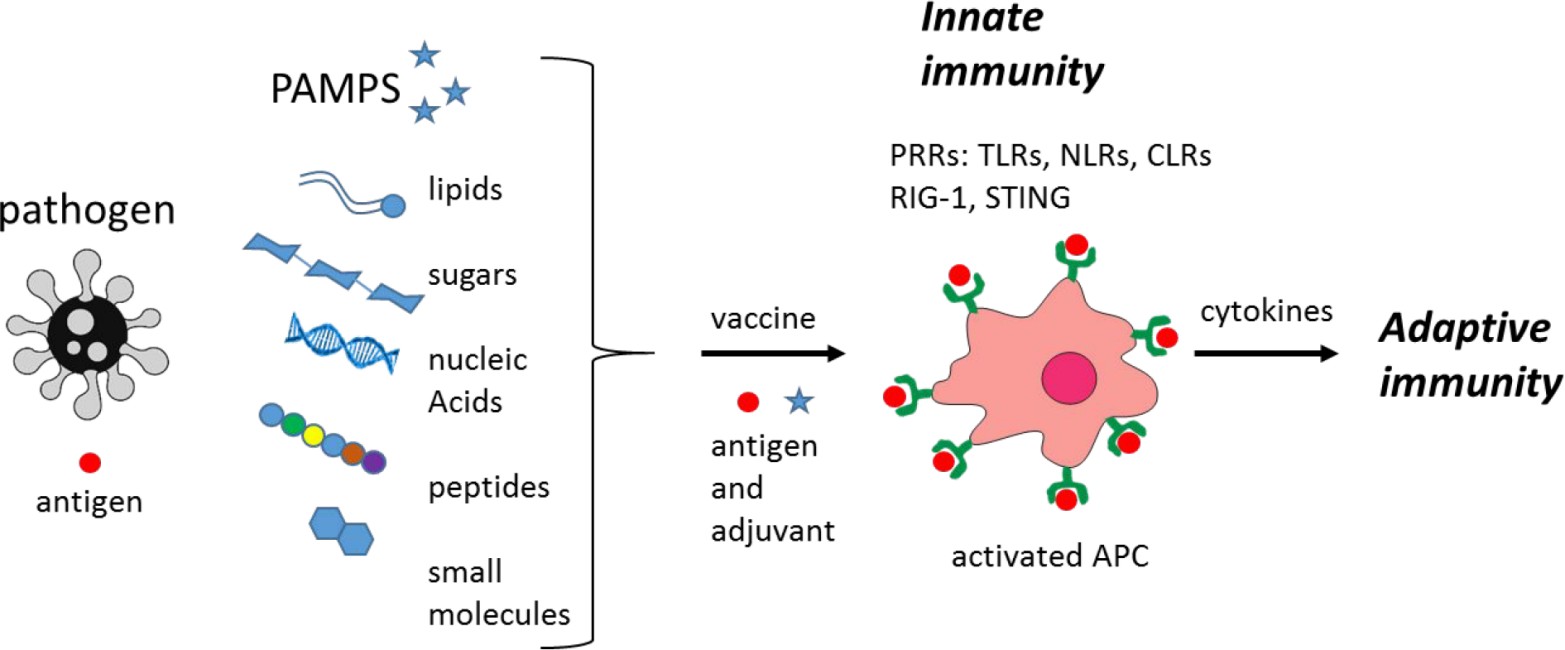
Innate versus adaptive immunity. The types of PAMPs that stimulate the innate immune response via PRRs such as those shown (defined in the text) are indicated.

The innate immune response (Figure 3) recognizes pathogen-associated molecular patterns (PAMPs) via pattern recognition receptors (PRRs). Types of PRRs include Toll-like receptors (TLRs), C-type lectin agonists (CLRs), RIG-I (retinoic acid-inducible gene I), NOD-like receptors (NLRs), stimulator of interferon (IFN) genes (STINGs) (Figure 3), and others.^54−56^

The activated adaptive immune system exploits antigen-recognizing B cells, T cells, dendritic cells, and antibodies. As noted above, the adaptive immune system produces T-helper cells, which release cytokines to assist other immune cells. T-helper cells differentiate into Th1 cells or Th2 cells. The cell-mediated response relies on the former, and the humoral response involves Th2 cells (Figure 2). This refers to the production of antibodies or antimicrobial peptides in extracellular fluid (and is also known as antibody-mediated immunity).

The activity of a vaccine may be improved using an adjuvant, which is an additive that stimulates a stronger immune response. These were traditionally based on inorganic materials, especially alum, but more recently, organic systems, especially emulsions and liposome formulations, have been developed. Lipopeptides, especially those containing the PamCS (palmitoyl-Cys-Ser) motif, can show self-adjuvant properties, as discussed elsewhere.^45,57^ As pointed out by Abudula et al., self-assembling peptides may have adjuvant activity that results from the formation of depots of antigens, by directing vaccines to APCs, or by the improvement of immune-cell priming.^58^ Organic vaccine adjuvants have been discussed in a number of reviews,^54,59,60^ and a review specifically focused on adjuvants for subunit-based peptide vaccines is available.^61^

This review is focused on the development of immunogenic peptides for applications in vaccines. This complements my recent review on lipopeptides for vaccine development,^45^ and the current overview excludes material previously covered, that is, discussion of lipopeptides as immunogens or adjuvants. It also does not cover potential peptide vaccines for neurodegenerative diseases such as Alzheimer’s disease, which has been recently reviewed.^25^ This review is focused on subunit vaccines based on unconjugated peptides. As this is a vast and also fast-moving field, the aim is to capture some key findings and concepts, and unfortunately it is not possible to cover all the exciting work on this subject.

This review is organized as follows. First in section 2, model peptide based antigens (and adjuvants) are discussed, in particular those based on self-assembling peptides. Then section 3 covers peptides for vaccines for a range of viral and other infectious diseases, including the highly topical subject of SARS-CoV-2 peptide-based vaccines. This is followed by section 4 on cancer immunotherapy peptides. Section 5 provides concluding remarks. Table 2 lists key peptide sequences discussed in this review.

**Table 2:** Key Peptide Sequences Highlighted in This Review

sequence	origin	application	refs
SIINFEKL	ovalbumin	model antigen	(62−71)
LPDEVSGLEQLESIINFEKLTEWTSSNVMEER	ovalbumin (longer sequence incorporating preceding)	model antigen	(69)
ISQAVHAAHAEINEAGR	ovalbumin	model antigen	(72)
SGPSNTPPEI	adenovirus Ad5 E1a protein	model antigen	(65)
LEEKKGNYVVTDH	B cell epitope from epidermal growth factor receptor class III variant	model antigen	(64)
AKXVAAWTLKAAA	pan HLA DR-binding epitope (PADRE)	model antigen	(64,73)
GQIGNDPNRDIL	universal Th cell epitope	model antigen from tetanus toxin	(74−76)
QYIKANSKFIGITE	universal Th cell epitope	model antigen from tetanus toxin	(74−76)
FNNFTVSFWLRVPKVSASHLE	universal Th cell epitope	model antigen from tetanus toxin	(74−76)
AQYIKANSKFIGITEL	Th epitope	model antigen from tetanus toxin	(77)
STDSCDSGPSNTPPEI	human adenovirus type 5 early region 1B CTL epitope	model antigen	(78)
QLINTNGSWHIN	HCV E2 envelope glycoprotein epitope I	HCV antigen	(79)
CGWVAGLFYYHKF	HCV E2 envelope glycoprotein epitope II	HCV antigen	(80)
LMGYIPLVGA	HCV core TCL epitope	HCV antigen	(81)
EGRAWAQPGYPWPLYGNEGL	HCV core Th epitope	HCV antigen	(82)
AVGIGAVFLGFLGAAG and AVGIGAVF	HIV envelope glycoprotein gp41 fragments	HIV antigen	(83)
LDKWASLWNWFNITNWLWYIR	HIV gp41 membrane proximal external region (MPER) epitope	HIV antigen	(84,85)
ELLELDKW	HIV gp41 MPER epitope	HIV antigen	(86)
RIQRGPGRAFVTIGK	HIV gp160 CTL epitope	HIV antigen	(87)
SLYNTVATL	HIV glycoprotein CTL epitope	HIV antigen	(88,89)
ILKEPVHGV	HIV Pol DNA polymerase CTL epitope	HIV antigen	(88)
KQIINMWQEVGKAMYA	HIV gp120 Th epitope	HIV antigen	(90)
NPNA (NANP) repeats	*P. falciparum* CS protein motif	malaria antigen	(25,91−94)
YLQPRTFLL	SARS-CoV-2 T cell epitope	SARS-CoV-2 antigen	(95)
FLLNKEMYL	SARS-CoV-2 T cell epitope	SARS-CoV-2 antigen	(95)
FIAGLIAIV	SARS-CoV-2 T cell epitope	SARS-CoV-2 antigen	(96)
FVSEETGTL	SARS-CoV-2 T cell epitope	SARS-CoV-2 antigen	(96)
YVYSRVKNL	SARS-CoV-2 T cell epitope	SARS-CoV-2 antigen	(97)
SLVKPSFYV	SARS-CoV-2 T cell epitope	SARS-CoV-2 antigen	(97)
LAILTALRL	SARS-CoV-2 T cell epitope	SARS-CoV-2 antigen	(97)
WTAGAAAYY	SARS-CoV-2 HLA-binding epitope	SARS-CoV-2 antigen	(98)
GAAAYYVGY	SARS-CoV-2 HLA-binding epitope	SARS-CoV-2 antigen	(98)
RSAIEDLLFDKV	common coronavirus spike protein sequence	SARS-CoV-2 and other coronavirus antigen	(99)
KRSFIEDLLFNKV	SARS cleavage site sequence	SARS-CoV-2 and other coronavirus antigen	(100,101)
ASTEK	SARS-CoV-2 RBD sequence	SARS-CoV-2 antigen	(102)
PKKS	SARS-CoV-2 RBD sequence	SARS-CoV-2 antigen	(102)
QLQMGFGITVQYGT	MERS B cell epitope	MERS-CoV antigen	(103)
YKLQPLTFL	MERS T cell epitope	MERS-CoV antigen	(103)
YCILEPRSG	MERS T cell epitope	MERS-CoV antigen	(103)
SVVNIQKEIDRLNEVAKNLN	SARS-CoV spike protein B cell epitope	SARS-CoV antigen	(104)
RPQASGVYMGNLTAQ	lymphocytic choriomeningitis virus (LCMV) nucleoprotein T cell epitope	LCMV antigen	(105,106)
HGEFAPGNYPALWSYA	murine respirovirus nucleoprotein epitope	murine respirovirus (Sendai virus) antigen	(107)
FAPGNYPAL	murine respirovirus CTL epitope	murine respirovirus (Sendai virus) antigen	(108−110)
CDSGPSNTPPEIHPVV	adenovirus type 5 E1A protein sequence	used in a murine respirovirus candidate vaccine	(110)
RGYVYQGL	vesicular stomatitis virus (VSV) nucleoprotein sequence	VSV antigen	(111,112)
RFKMFPEVKEKGMAG	human glutamic acid decarboxylase (GAD)65 protein T cell epitope	insulin-dependent diabetes mellitus (IDDM) antigen	(113)
FTSEHSHFSL	human glutamic acid decarboxylase (GAD)65 protein T cell epitope	insulin-dependent diabetes mellitus (IDDM) antigen	(113)
KIFGSLAFL and KIFGSLAFLPESFDGDPA	minimal HER-2 epitope	HER-2 cancer antigen	(114,115)
IISAVVGIL	HER-2/neu protein fragment	HER-2 breast cancer antigen	(116,117)
GVGSPYVSRLLGICL	HER-2/neu protein fragment	HER-2 breast cancer antigen	(118,119)
PESFDGDPASNTAPLQPEQLQ	HER-2 antibody binding peptide	HER-2 breast cancer antigen	(120)
YMPIWKFPDEEGAC	HER-2 antibody binding peptide	HER-2 breast cancer antigen	(120)
CRVLQGLPREYVNARHC	HER-2 antibody binding peptide	HER-2 breast cancer antigen	(120)
VAR**C**PSGVKPDLSYMPIWKFPDEEGA**C**QPL (**C**: disulfide crosslink site)	HER-2 peptide sequence	HER-2 breast cancer antigen	(121)
KIFGSLAFLPESFDGDPA	HER-2 peptide sequence	HER-2 breast cancer antigen	(115)
RRLLQETELVEPLTPS	HER-2 peptide sequence	HER-2 breast cancer antigen	(115)
HGVTSAPDTRPAPGSTAPPA	variable number of tandem repeats (VNTR) domain of MUC1 B cell epitope	MUC1 cancer antigen	(76,122)
VLSNDVCAQV (and VISNDVCAQV)	prostate-specific antigen (PSA) epitope	prostate cancer	(42,123,124)
ALDVYNGLL	prostatic acid phosphatase (PAP) peptide sequence	prostate cancer	(125)
ALQPGTALL	prostate steam cell antigen (PSCA) sequence	prostate cancer	(126)
EIWTHSTKV	folate receptor-α sequence	ovarian cancer antigen	(25,77)
MHTAPGWGYRLS	folate receptor-α sequence	ovarian cancer antigen	(127)
SLLMWITQCFLPVF (and SLLMWITQC)	antigen derived from NY-ESO-1 containing both Th and Tc epitopes	antigen expressed in a number of cancers	(41,128)
LLEFYLAMPFAT	NY-ESO-1 epitope	antigen expressed in a number of cancers	(129)
IMDQVPSFV	modified melanoma differentiation glycoprotein gp100 sequence (cf. preceding entry)	melanoma antigen	(130)
SSPGCQPPA	melanoma differentiation glycoprotein gp100 sequence	melanoma antigen	(131)
YMDGTMSQV	tyrosinase sequence	for melanoma vaccine	(130)
QCSGNFMGF	tyrosinase sequence	for melanoma vaccine	(131)
LHHAFVDSIF	tyrosinase sequence	for melanoma vaccine	(131)
TWHRYHLL and TAYRYHLL	tyrosinase gp75 protein sequence and variant	for melanoma vaccine	(132)
AAAPKIFYA	melanoma CTL epitope from screening	melanoma antigen	(133)
KASEKIFYV	melanoma CTL epitope from SSX protein	melanoma antigen	(133)
KYICNSSCM	p53 tumor antigen protein sequence	p53 tumor antigen	(134)
LGFLQSGTAKSVMCT	P53 Th epitope	p53 tumor antigen	(135)
FEQNTAQP	murine lung tumor-associated antigen peptide	murine lung carcinoma antigen	(136,137)
FEQNTAQA	murine lung tumor-associated antigen peptide	murine lung carcinoma antigen	(136,137)
AAGIGILTV and EAAGIGILTV	Melan-A-specific CTL peptides	melanoma antigen	(138,139)
LAGIGILTV	Melan-A-specific CTL peptide variant (cf. preceding)	melanoma antigen	(140)
CYTWNQMNL	Wilm’s tumor gene modified CTL epitope	Wilm’s tumor antigen (associated with some leukemias and others)	(141)
SSIEFARL and SEIEFARL	herpes simplex virus glycoprotein sequence and modification	model viral tumor antigen	(132)
RAHYNIVTF	HPV E7 protein CTL epitope	HPV-induced tumors	(69,142,143)
QAEPDRAHYNIVTFCCKCDSTLRLCVQSTHVDIR	HPV E7 protein CTL epitope	HPV-induced tumors	(69)
MDRVLSRADKERLLELLKL	polyoma virus T-antigen	polyoma virus-induced tumors	(144)
EPLTSLTPRCNTAWNRLKL	murine leukemia virus (MuLV) CTL epitope	MuLV-induced tumors	(145)
SSWDFITV	murine leukemia virus (MuLV) CTL epitope	MuLV-induced tumors	(145)
SPSYVYHQF	murine leukemia virus (MuLV) gp70 protein CTL epitope	MuLV-induced tumors	(145)
LPYLGWLVF	murine mastocytoma P815 cells	tumor antigen	(62)

## 2 Model Self-Assembling Peptide Antigens and Adjuvants

Sequences from ovalbumin (OVA) have been used as model antigens. These can stimulate CD8^+^ T cell responses as demonstrated, for example, in a study combining an ER (endoplasmic reticulum) insertion sequence signal peptide (RYMILGLLALAAVCSAM) with epitopes from chicken ovalbumin (SIINFEKL, amino acids, aa 257–264) or a natural tumor antigen expressed by the murine mastocytoma P815 (P1A aa 35–43, LPYLGWLVF).^62^ Immunization with the fusion peptide RYMILGLLALAAVCSAMSIINFEKL significantly extended the survival of mice challenged with a thymoma (cancerous thymus) transfected with the complementary DNA of chicken ovalbumin.^62^ Sequences from OVA, especially SIINFEKL, have been widely used as model immunogens as discussed in the following examples.

**Figure 4:**
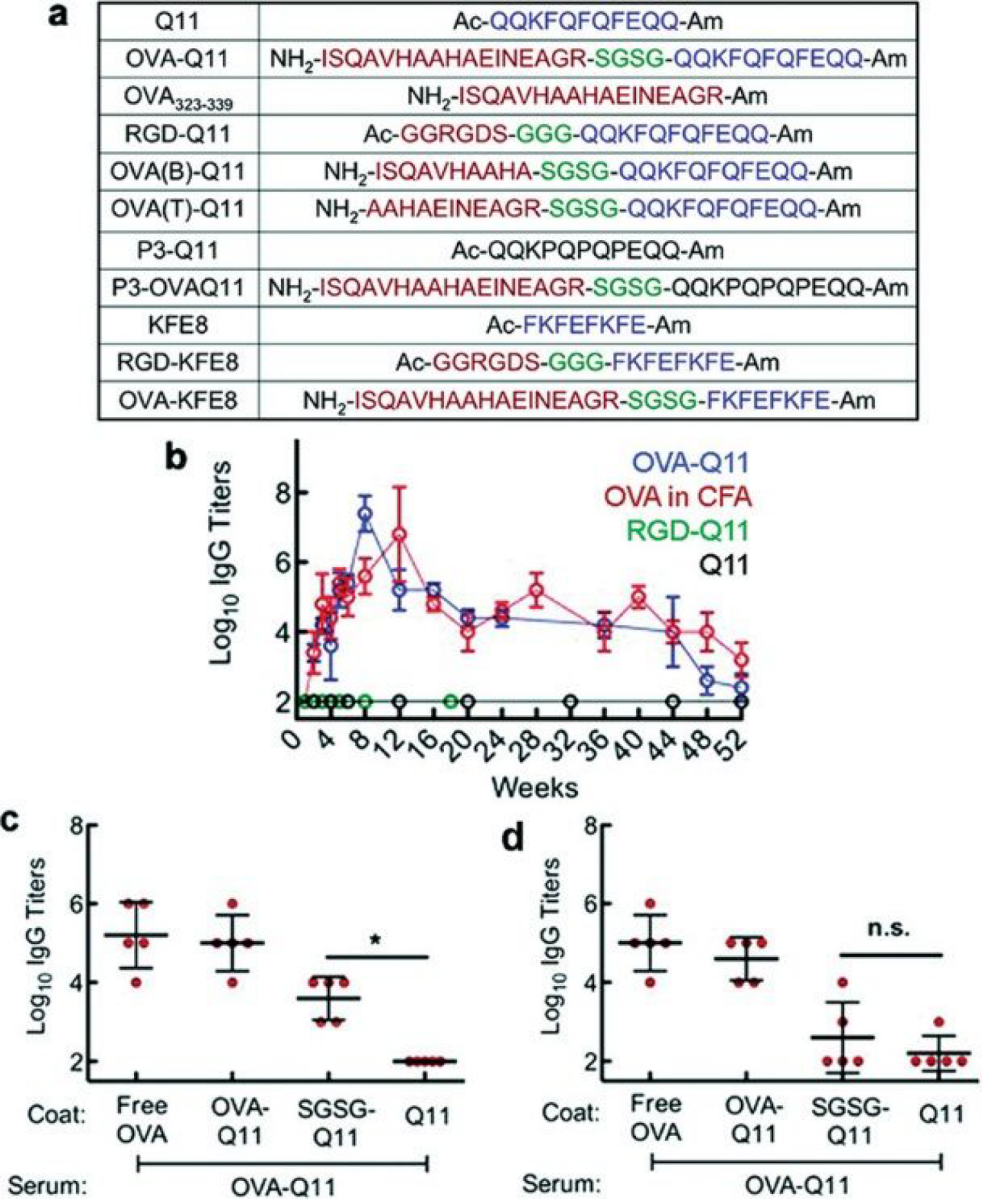
(a) Self-adjuvant peptide sequences studied by Collier’s group based on the Q11 fibrillizing peptide (blue sequence and non-fibrillizing proline variants, black sequences) and sequences from ovalbumin OVA323-339 (red sequence) with a short hydrophilic spacer (green). (b) Long time scale antibody response, comparing Q11 hybrids with the Q11 peptide and the OVA sequence in CFA (complete Freund’s adjuvant), after initial dose and half-initial dose booster after 4 weeks. (c, d) ELISA antisera analysis of sera after 5 weeks (c) or 24 weeks (d). *p < 0.05 by ANOVA using Tukey post hoc test. Reproduced from ref (146). Copyright 2012 American Chemical Society.

A peptide, Q11 (QQKFQFQFEQQ), that forms β-sheet fibrils has been used as a platform to display biologically active motifs, including the RGD tripeptide and model antigens.^63,72,146^ The Q11 peptide by itself or with complete Freund’s adjuvant (CFA, an emulsion containing inactivated mycobacteria) is non-immunogenic. However, linking a sequence from ovalbumin (OVA, chicken egg ovalbumin sequence 323–339, ISQAVHAAHAEINEAGR, Figure 3a) was shown to lead to the production of antibodies (immunoglobulin titers, Figure 3b–d) in mice, without the need for additional adjuvant (i.e., it is self-adjuvating).^72^ This ovalbumin domain contains both T and B cell epitopes and was linked to Q11 via a short SGSG hydrophilic spacer (Figure 3a). OVA stimulates CD40-driven T and B cell responses.^147^ The immune response was suggested to be dependent on self-assembly since, like the parent Q11 peptide, Q11–OVA forms β-sheet fibrils and a variant peptide with three F → P substitutions (Figure 3a), which does not fibrillize, also does not raise antibodies.^72^ In fact self-assembly and conformation were studied in PBS buffer solution, not under *in vivo* conditions, and it was not established whether the conjugates form β-sheet fibrils under these conditions. The antibody response was found to be T cell-dependent, and no notable antibody stimulation was observed for Q11 conjugates to OVA fragments comprising only B or T cell epitopes (sequences shown in Figure 4a).^146^ Subsequently, the low cytotoxicity and inflammatory properties of this conjugate were investigated in more detail, in comparison to the conventional adjuvant alum, the results demonstrating low cytotoxicity (analysis of tissue swelling and cellular and cytokine responses).^148^ Immunization with nanofibers bearing epitopes led to differentiation of T cells into T follicular helper (Tfh) cells and of B cells into germinal center cells in an antigen-specific manner and produced IgG that was neutralizing in influenza hemagglutination inhibition assays and cross-reacted with the native protein antigen. Increased expression of the CD80 and CD86 activation markers (of dendritic cells) was observed in the presence of peptide nanofibers.^148^

The adjuvant activity of an alternative fibrillizing peptide, KFE8 (FKFEFKFE) in a conjugate with OVA was also shown since a KFE8–OVA hybrid generates an immune response (IgG titers) while the parent KFE8 peptide does not. The authors point out that the similar immunogenicity of the two very distinct peptide–OVA constructs indicates the lack of sensitivity to fibrillizing peptide sequence.^146^ The group later showed that a conjugate of Q11 to the OVA sequence OVA_257–264_, SIINFEKL, stimulates a response of CD8^+^ T cells, which is desirable for effective adjuvant activity (note that others have suggested that such short sequences do not stimulate CD8^+^ T cell responses).^63^ The authors highlighted the advantage of the system as a non-inflammatory system that can be stored at room temperature, eliminating the need for cold chain storage.^63^ The KFE8 peptide has also been used in a mixture with West Nile Virus (WNV) EIII receptor-binding domain from the envelope protein.^149^ An emulsified mixture of the KFE8 peptide adjuvant hydrogel and the EIII protein was shown to produce robust antibody responses and to confer significant protection in the mouse model against lethal infection.^149^

The same OVA peptide, SIINFEKL, was developed earlier as a model antigen in a study of the effect of combination of a peptide immunogen with a TLR agonist.^65^ The peptide was delivered transcutaneously in the form of an ointment containing the TLR7 agonist imiquimod,. The use of a transdermal delivery method to prime CTLs and the full immune response observed (in the mouse model employed) are interesting aspects of the work. The peptide SGPSNTPPEI (SGP) from the adenovirus Ad5 E1a protein (aa 234–243) also generates a CTL response, peptide and imiquimod both being required to prime a T cell response.^65^ This epitope had previously been shown to prime CTL cells in a vaccine with IFA (incomplete Freund’s adjuvant) and an activating monoclonal antibody to promote CD40 activation.^150,151^ This latter is essential for the induction of therapeutic CTL immunity using a tumor-specific peptide vaccine in tumor-bearing mice.^150^ Peptide SIINFEKL is sufficiently widely used as an OVA antigen that cells (B3Z hybridomas) responsive via TCRs to this sequence are available.^66^ However, it has been shown that serum proteases can disrupt presentation of SIINFEKL by MHC class I molecules due to proteolysis.^67^ On the other hand, the presentation of the full OVA sequence can be enhanced in the presence of β_2_-microglobulin in serum. This can be blocked using appropriate protease inhibitors (in this case an aminopeptidase inhibitor but not an endopeptidase inhibitor), and the authors also point out that minimal sequences such as SIINFEKL may need modification or extension to guard against serum inactivation.^67^ Degradation by peripheral DCs has been noted for other short peptide antigens.^139^

The SIINFEKL motif has been incorporated in synthetic vaccines comprising this sequence linked to either the TLR9 DNA ligand, CpG, or the TLR2 ligand Pam_3_CysSK_4_.^68^ Fast, enhanced uptake of both types of TLR-conjugated peptides was observed in DCs, although the uptake mechanisms were distinct.^68^ Pam_3_CSK_4_ and related lipopeptides are discussed in recent reviews on lipopeptides for vaccine development.^45,57^

The preceding examples of β-sheet forming peptides and many others in Table 2 contain aromatic residues, which can promote fibril formation due to π-stacking interactions.^152−154^ In fact, the examples in Table 2 indicate a prevalence of aromatic residues above that typically found in proteins (<10% for F, W, and Y together^13^). However, many epitopes in Table 2 do not form β-sheet structures, and the aromatic residues may play important roles in interactions with particular receptors. Coiled-coil constructs have been investigated as model peptide assemblies potentially able to stimulate immune responses. In one example, a sequence from the coiled-coil domain of the γ-chain of mouse fibrinogen was used as a template to create a related coiled-coil forming peptide and a triblock of this peptide with a central PEG chain.^155^ The parent peptide had an unordered conformation; however the derivative and peptide–PEG–peptide triblock had similar high helical content of secondary structure based on CD spectra. The distribution of aggregates present was probed using analytical ultracentrifugation, which revealed the presence of dimers and tetramers or pentamers for the peptide and predominantly dimers for the triblock, along with a population of larger multimers (up to 50-mers). Only the triblock raised antibodies in mouse serum; however there was no evidence for T cell production by splenocytes or lymph node cells.^155^ In contrast to the effective β-sheet fibril conjugates developed by the same group that show T cell responsiveness, on the basis of these results further research on the coiled-coil systems was not pursued.

In another example of a vaccine platform based on coiled-coils, model antigens including SIINFEKL, a PADRE epitope (section 3.3, aKXVAAWTLKAa), or the epidermal growth factor receptor class III variant B cell epitope LEEKKGNYVVTDH were attached at the N-terminus of a model 29-residue coiled-coil-forming peptide.^64^ These peptides aggregated into fibrils, which were internalized by APCs and generated robust antibody and CD4^+^ and CD8^+^ T cell responses in mice, without supplemental adjuvants.^64^

## 3 Peptides for Vaccines for Infectious Diseases

### 3.1 Influenza

Human influenza pandemics were responsible for between 50 and 100 million deaths in the last century.^156^ The development of effective vaccines for influenza is challenging due to the huge sequence diversity and high mutation rate of influenza viruses. One target is influenza hemagglutinin (HA), a family of glycoproteins that enable viral entry into host cells. These glycoproteins exhibit substantial variation in their sequence and glycosylation patterns, which are important strategies to escape host immune responses.^157^ Despite this, it has been possible to isolate broadly neutralizing antibodies against these viruses, and the structure of these antibodies has been investigated. The thousands of influenza A strains fall into two major groups and can be further classified into 17 HA subtypes according to their reactivity against polyclonal antisera.^157^ HAs are shuffled into a circulating human virus from the huge reservoir of HA subtypes in avian viruses in order to evade immunity within the population. To attempt to circumvent sequence diversity, vaccine design has focused on highly conserved domains, especially those of viral envelope glycoproteins that are targeted by broadly neutralizing antibodies.^25^ Since the “stem” region of hemagglutinin HA2 is highly conserved, it represents an excellent target.^158^ Based on the stem, “mini-HAs” (molecular weight 40–242 kDa) were developed, and the best candidate exhibited structural and broadly neutralizing antibody binding properties similar to those of full-length HA and was shown to protect mice after exposure to influenza and to reduce fever in monkeys after sublethal challenge.^158^ The structural features of antibodies that bind to the HA stem have been investigated, and this has led to the identification of some conserved residues.^156,159^

**Figure 5:**
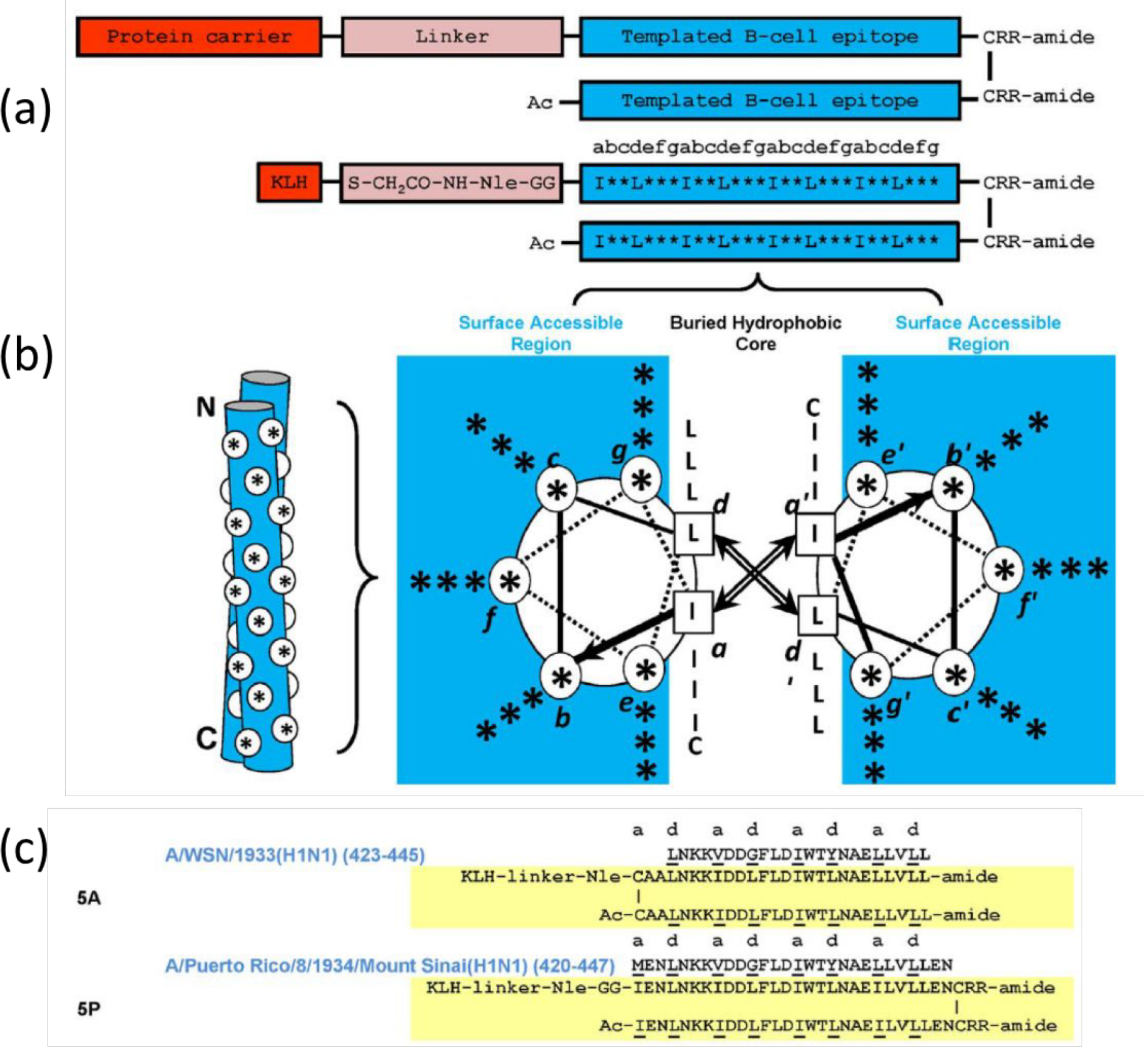
Coiled-coil peptide constructs that present HA sequences.^161^ (a) Construct design, (top) schematic, (bottom) detail. (b) Dimeric coiled-coil showing noninterface residues as *. The knobs-in-holes packing is shown on the left (N and C termini indicated), while the hydrophobic interactions between L and I residues at positions a and d are shown in the helical wheel representation on the right (heptad positions abcdefg shown), (c) Specific sequences of two of the peptides studied including 5P with the highest cross-reactivity. Reproduced with permission from ref (161). Copyright 2016 Wiley-VCH.

Synthetic peptides that contain fragments of HA2 are able to elicit antibody titers. Wang et al. showed that a mouse vaccine containing a HA2-based synthetic peptide protects against influenza viruses of subtypes H1N1, H3N2, and H5N1, which diverge in structure.^160^ Based on earlier work on the H3 subtype virus, they used the long α-helical (LAH) sequences, residues 76, 130, of HA2. A conjugate vaccine was synthesized that comprises the LAH sequence and a C-terminal spacer domain of eight amino acids (a so-called Flag tag) followed by a cysteine residue to enable coupling to the carrier protein keyhole limpet hemocyanin (KLH). The conjugate may bind residues within a single α-helical portion of the HA2 protein.^160^ Hodge’s group produced an immunogen that produces antibodies to group 1 or group 2 HAs, depending on the sequence.^161^ This group used *de novo* principles to design a double stranded α-helical coiled-coil template that contains conserved α-helical epitopes from the region of the stem of influenza A HA glycoproteins. The construct, shown in Figure 5, also contains a KLH carrier attached via a spacer to the cysteine-linked coiled-coil region, stabilized by patterned hydrophobic I and L residues, consistent with coiled-coil design principles (and two arginine residues are included to improve solubility). The actual peptide sequences are also shown in Figure 5. The immunogen 5P demonstrates the strongest cross-reactivity against group 1 and group 2 HA proteins.^161^

Multimeric-001 is a peptide vaccine for influenza that has proceeded to stage III clinical trials, being based on both B and T cell (CTL and Th) epitopes from HA, nucleoprotein (NP), and matrix 1 (M1) and sequences combined as triplicates within a single recombinantly expressed polypeptide.^26−28^ The sequences are shown in Table 3. This recombinant peptide can be produced using standard fermentation procedures and can be readily deployed for human use.^27^ Multimeric-001 can be used as a complete vaccine or as a primer for a H5N1 influenza vaccine.^28^ As expected since it has reached phase III trials, Multimeric-001 is effective against a variety of strains as a separate vaccine or as a pandemic primer, and it has a good safety profile.

**Table 3:** Sequences of the Components of Peptide Influenza Vaccine Multimeric-001^26^

peptide^a^	amino acid sequence
HA epitope 1	PKYVKQNTLKLAT
HA epitope 2	SKAYSNCYPYDVPDYASL
HA epitope 3	WLTGKNGLYP
HA epitope 4	WTGVTQN
HA epitope 5	PAKLLKERGFFGAIAGFLE
NP epitope 6	FWRGENGRKTRSAYERMCNILKGK
NP epitope 7	SAAFEDLRVLSFIRGY
NP epitope 8	ELRSRYWAIRTRSG
M epitope 9	SLLTEVETYVP

a HA, hemagglutinin; NP, nucleoprotein; M, matrix protein. The peptide sequence is (HA epitope 1) - (HA epitope 2) - (M epitope 9) - (HA epitope 3) - (HA epitope 4) - (NP epitope 6) - (HA epitope 5) - (NP epitope 7) - (NP epitope 8).

A candidate influenza vaccine able to protect mice has been developed based on VLPs originating from the RNA bacteriophage AP205.^162^ This scaffold was shown to provide a versatile carrier for a variety of peptide epitopes. Peptides derived from angiotensin II, CXCR4 receptor, *Salmonella typhi* outer membrane protein, gonadotropin releasing hormone (GnRH), or influenza A M2 protein were linked to either terminus of the AP205 coat protein, and some were able to generate peptide-specific antibodies. In particular, the VLPs containing the influenza-related peptide generated a protective immune response, generating IgGs and lengthening the survival of mice.^162^ A vaccine against avian influenza that is based on an extended coiled-coil peptide that aggregates into polyhedral virus-like particles has been tested in chickens.^163^ These are icosahedral or octahedral, respectively, for 97-residue peptides designed to form pentameric–trimeric coiled-coils or tetrameric coiled-coils. The tetrameric construct with adjuvant (complete or incomplete Freund’s adjuvant) offered protection against one flu subtype, H5N2.^163^

*In silico* methods (ClustalW sequence analysis and prediction of immunogenicity) were used to identify T cell epitopes for influenza A and B.^164^ The six identified T cell epitopes were then synthesized, and four lead candidates were examined as a potential influenza vaccine mixture. The induction of a HLA-specific Th1-like immune response was examined. The survival of transgenic mice against lethal challenge with influenza was significantly enhanced by immunization.^164^ This vaccine (Flu-v) has progressed to stage II clinical trials.^165^

Another candidate vaccine in which conserved B- and T-cell epitopes are combined is VaccFlu. The peptides in the mixture employed were developed using a proprietary platform based on responses to HLA-restricted epitopes.^166^ Wild-type and transgenic HLA-A*02:01 mice immunized with the peptide mixture showed both cell and humoral immune responses, and the vaccine can provide protection from severe disease symptoms upon infection.^166^

### 3.2 Hepatitis C and Hepatitis B

Hepatitis C virus (HCV) infections can cause liver diseases such as cirrhosis or hepatocellular carcinoma. Both CD4^+^ and CD8^+^ T cells are involved in the response to infection, and the role of the humoral immune system has been highlighted.^167^ There are currently no vaccines for this condition, although trials of candidates are underway.

Development of a hCV vaccine that is effective has been hindered by the variability of the virus, resulting from mutations that facilitate circumvention of the immune system, specifically sequence variation within epitopes targeted by T cells.^25,168^ The properties of HCV have been compared to those of other hepatitis viruses, for which vaccines are available.^169^ Broadly neutralizing antibodies (bnAbs) can even abrogate pre-existing infection,^170^ and the determinants for B cell response have been uncovered.^171^ The targeting by neutralizing antibodies of epitopes of HCV envelope glycoproteins has been discussed.^167^ A novel peptide vaccine, IC41, has been developed that comprises five synthetic peptides containing HCV T cell epitopes with adjuvant poly(l-arginine).^172^ Immunogenicity was assessed by examining T cell epitope-specific [^3^H]-thymidine proliferation and IFN-γ and using HLA tetramer binding assays, and these studies confirmed that IC41 was well tolerated and that it induces Th1 and CTL responses in all dosed groups.^172^ However, on further examination it was found that T cell responses were too small to produce significant differences in HCV RNA for most patients, so further optimization is needed.^173^ The authors also noted that the peptide vaccine may also have restricted utility, since only a minority of possible epitopes are included, and repeated stimulation with a small number of peptides may narrow the CTL response. Later, it was shown that an improved dose regimen or intradermal injection can be used to improve the immunogenicity of IC41.^174^ Topical application of the TLR7 agonist imiquimod did not enhance immunogenicity. In a phase II clinical trial, a modest, but not clinically meaningful, decrease in viral load was noted in patients receiving IC41 (with topically applied imiquimod); however, HCV viral load reduction and T cell immune response were not found to be correlated.^175^ It was thus proposed that these studies provide proof-of-principle as a basis for further research, for example, on combination therapies with antiviral drugs.^175^

**Figure 6:**
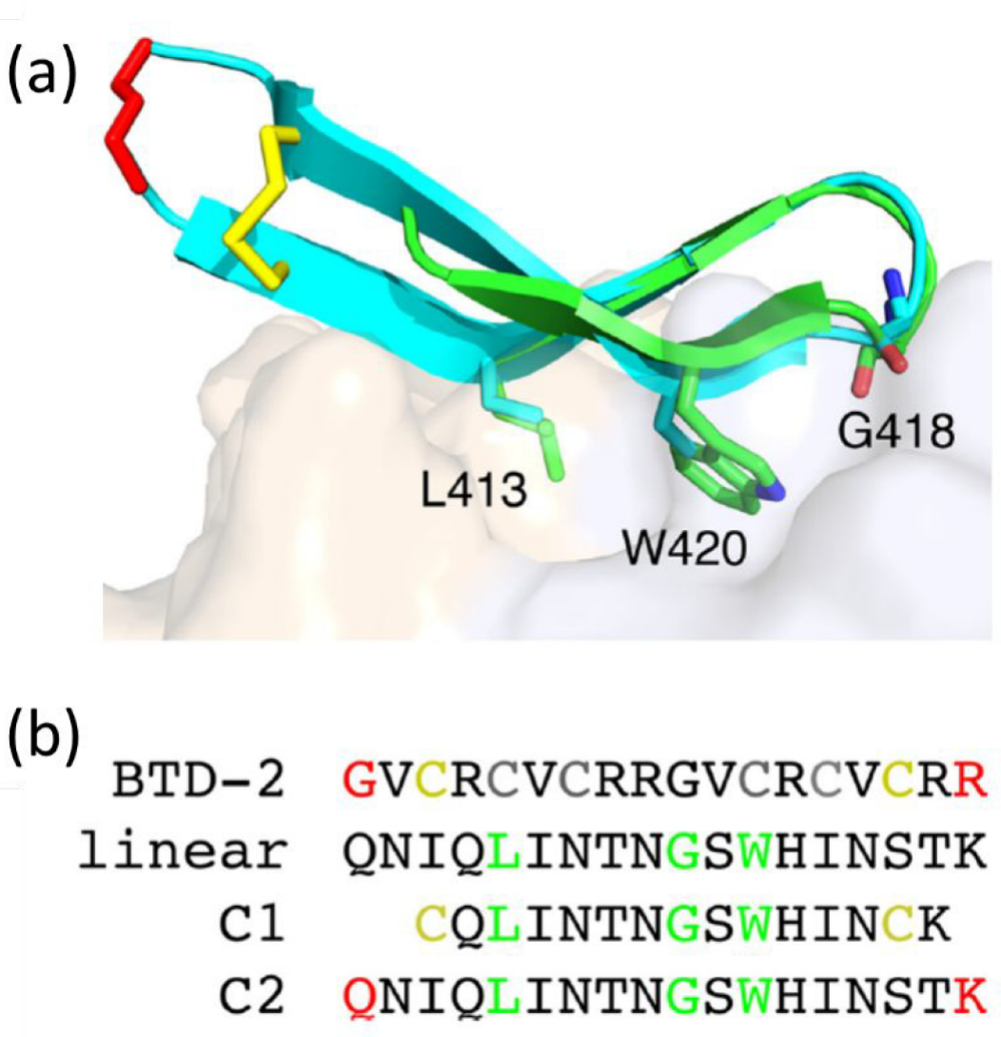
(a) Alignment of HCV-bound E2 epitope I (PDB code 4DGY; green) with a conformer of a cyclic θ-defensin peptide BTD-2 obtained from NMR (PDB code 2M2S; cyan), sticks represent cyclized residues (yellow, disulfide bridge; red, locations of backbone cyclization) and key epitope positions L413, G418, and W420. (b) Sequences of peptides including peptide C1 cyclized via disulfide linkages of yellow cysteine residues and C2 via the red residues (cf. BTD-2). Reproduced with permission from ref (79). Copyright 2017 American Society for Microbiology.

The majority of antibodies raised against HCV react against E2 glycoprotein epitopes. Many antibodies recognize overlapping epitopes, and sequences of these have been obtained.^176,177^ Structure-based design principles were used to develop immunogens that stimulate antibody responses to the HCV E2 envelope glycoprotein (residues 412–423, QLINTNGSWHIN) epitope I.^79^ This led to constructs with a conserved linear epitope, in particular peptides based on a cyclic defensin protein (Figure 6) and an immunogen with two copies of this epitope at the E2 surface. Vaccination of mice with these peptides elicited antibody responses to epitope I, and the obtained mouse serum is able to neutralize HCV. It was noted that the cyclic designs produce enhanced epitope-specific responses and neutralization compared to the native peptide.^79^

**Figure 7:**
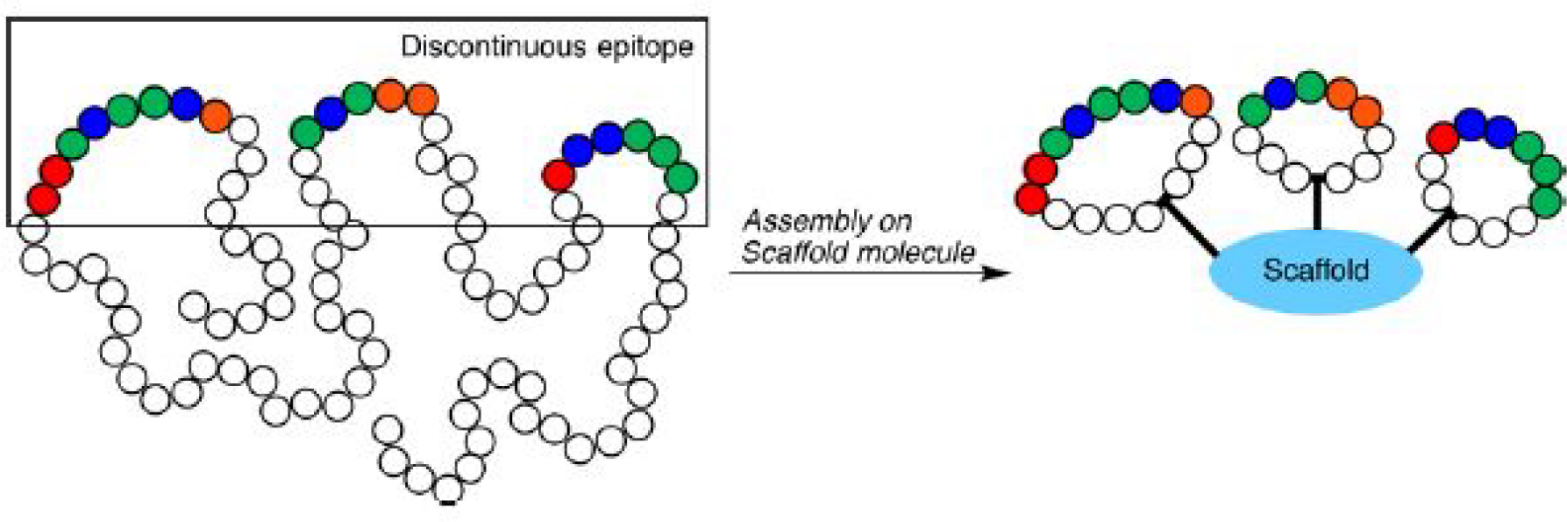
Epitope mimicry, in which discontinuous exposed fragments are displayed on a scaffold (here surface tethered molecules). Reproduced from ref (80). Copyright 2018 American Chemical Society.

Epitope mimicry is a concept in which discontinuous exposed epitope fragments are displayed on a scaffold as shown schematically in Figure 7.^80^ This approach was followed in the design of immobilized fragments of the HCV-envelope E2 protein. Thiol groups were used to covalently link the linear and cyclic epitope mimics on maleimide-activated plate surfaces.^80^ These constructs incorporated peptide antigen sequences based on epitope II of the HCV E2 glycoprotein, i.e. precursor peptide CGWVAGLFYYHKF. It was found that in contrast to linear epitope mimics, cyclic peptides showed specificity toward monoclonal antibodies targeted to HCV E2 epitope II. This *in vitro* system was used for diagnostic testing of antibody recognition using peptide-functionalized ELISA plates, which can be used for further enhancement of epitope design for vaccine development.^80^

Other approaches to creating HCV vaccines have been explored. Filskov et al. used a mixture of peptides that span the sequence of HCV nonstructural protein 3 (NS3) to present T cell epitopes.^178^ Broadened CD4^+^ and CD8^+^ T cell responses were observed in vaccinated mice using a panel of 62 20-residue peptide epitopes spanning the NS3 sequence. In another example, peptide-based subunit vaccines have been investigated, in particular the effect on CTL generation comparing vaccines based on Th or CTL epitopes of the HCV core, a mixture of CTL and Th peptides or a conjugated Th–CTL peptide.^179^ The peptides studied were the HCV core CTL epitope (C7A10; LMGYIPLVGA, aa 133–142),^81^ the Th epitope (CP4; EGRAWAQPGYPWPLYGNEGL aa 72–91)^82^ and the conjugated Th-CTL peptide (CP4–C7A10, EGRAWAQPGYPWPLYGNEGLLMGYIPLVGA). Mice immunized with C7A10, the C7A10/CP4 mixture or CP4-C7A10, but not those immunized with Th peptide alone, produced HCV core CTL epitope-specific effector cells.^179^

Hepatitis B virus (HBV) causes chronic hepatitis B, which is responsible for liver disease. A vaccine is now routinely available, which contains genetically engineered hepatitis B surface antigen (HBsAg). Another recently introduced vaccine, Heplisav, also targets HBsAg but also incorporates a TLR9 agonist adjuvant.^180^ A review on HBV vaccines is available.^181^

Candidate HBV peptide vaccines have recently been investigated. The B cell epitope HBsAg (113–135) has been displayed on a novel chimeric VLP carrier based on a bat HBV core antigen.^182^ The carrier was additionally optimized by incorporating one CD8^+^ T cell epitope and two CD4^+^ T cell epitopes. The resulting construct stimulates an antibody response specific to HBsAg (113–135), with increased T cell stimulation. In addition, lasting suppression of HBsAg and HBV DNA in HBV transgenic mice was noted.^182^ Immunotherapy with a recombinant vaccine comprising grass pollen antigen peptides and an HBV envelope protein domain can also produce antibody responses protecting against hepatitis B infection (see also section 3.6).^183^ HBV 15-mer peptide T cell epitopes that bind HLA class II alleles have been predicted using *in silico* methods.^184^ Sette et al. measured peripheral blood lymphocyte levels of patients with acute hepatitis to probe the antigenicity of *ca*. 100 different HBV-derived potential epitopes, all carrying HLA-A*02:01 binding motifs and found that an immune response is elicited above a defined affinity threshold.^185^

### 3.3 HIV

Human immunovirus (HIV) causes AIDS (acquired immune deficiency syndrome), a potentially lethal human disease. There are now treatments, mainly based on small molecule antiretroviral compounds, which can almost completely ameliorate the effects of the condition. For example, a 36-residue peptide, enfuvirtide (trade name Fuzeon), which inhibits the fusion of the gp41 HIV viral coat protein with cell membranes, preventing the virus from entering the cell, is available as a clinical treatment.^186^

Despite progress in the development of small molecule antiviral treatments, there is still considerable research interest in prophylactic vaccines since none have yet been brought into practice.^187^ The V3 (variable region 3 of the HIV envelope) loop of the HIV-1 virus spike membrane glycoprotein gp120^188^ represents a target for neutralizing antibodies and consequently has been identified as a promising candidate for peptide-based vaccine design.^25,189−192^ This glycoprotein is vital for viral infection as it enables HIV entry into the host cell. It was used in the development of AIDSVAX gp120 B/E from VaxGen, and later RV144, which combines AIDSVAX with ALVAC-HIV (vCP1521, from Sanofi-Pasteur), a recombinant canary pox priming immunogen.^193−195^ AIDSVAX was not successful after phase III trials^196,197^ in the US, while RV144 was the subject of further trials in Thailand but has not been approved due to limited efficacy.^187,194^

**Figure 8:**
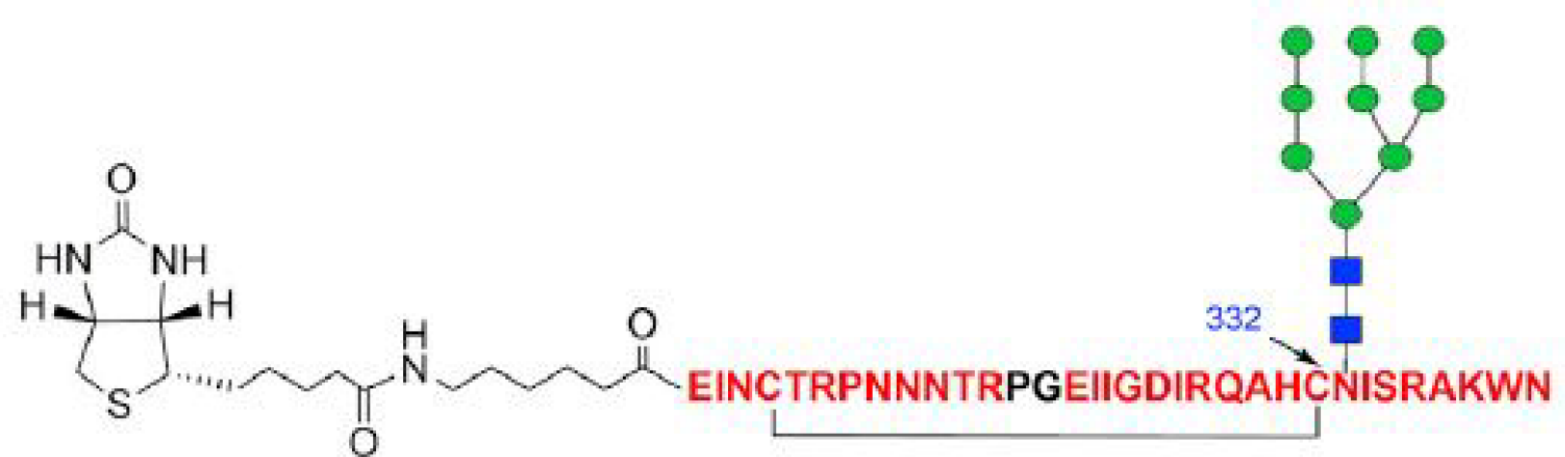
The 33-residue V3 glycopeptide epitope bearing a high-mannose glycan (green and blue are mannose and GlcNAc units, respectively) at the N332 site.^198^ The cyclization via disulfide formation from two cysteines is shown. The peptide is N-terminally biotinylated to facilitate site-specific immobilization for binding analysis. The sequence corresponds to a truncated V3 sequence containing residues 293–304 and 321–339, the tip residues 304–320 being replaced with a Pro-Gly dipeptide insert (black), which induces a reverse turn conformation. Reprinted from ref (198) with permission of Elsevier.

**Figure 9:**
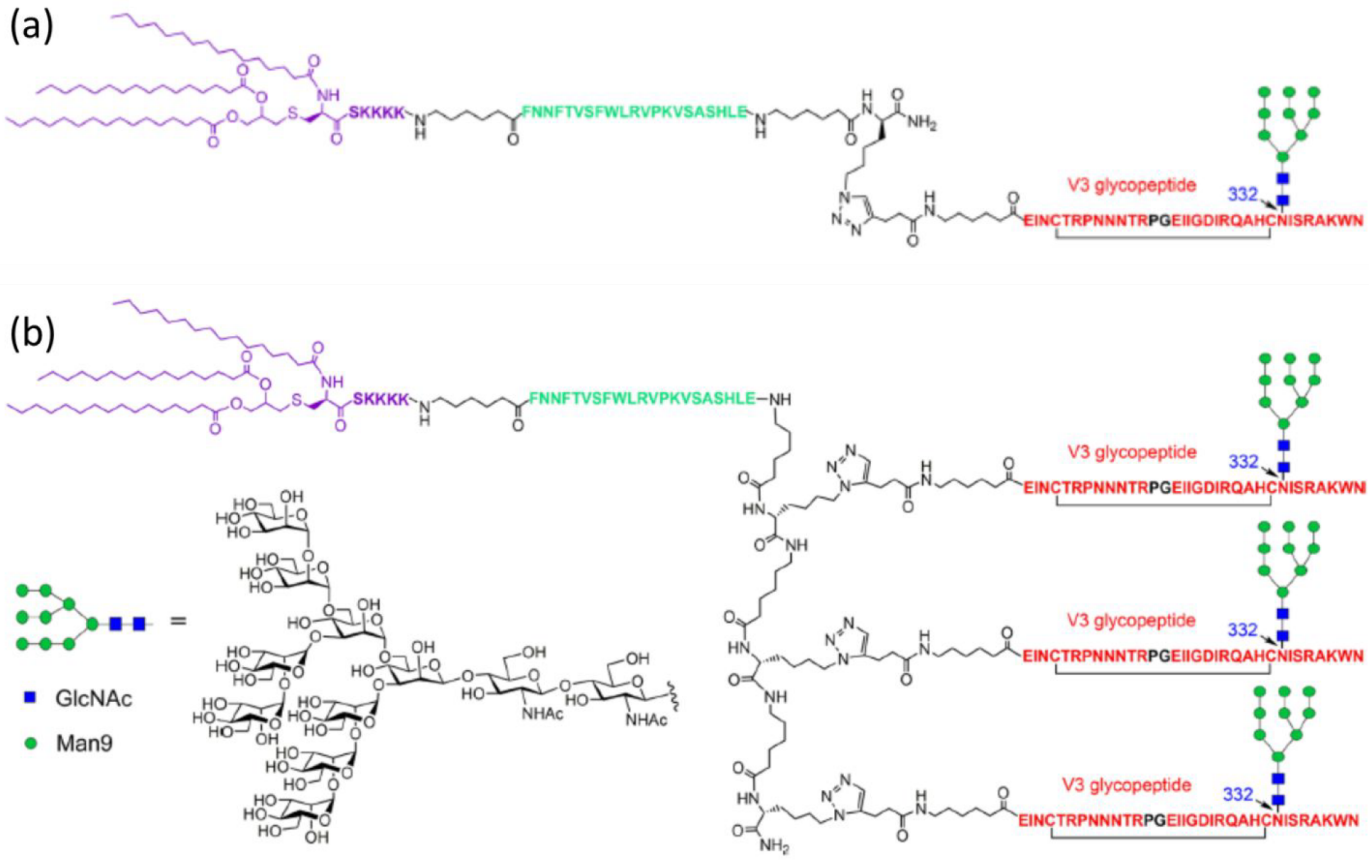
(a) Linear three-component conjugate containing a Pam_3_CSK_4_-based region (purple), a T-helper epitope (green), and the V3 peptide epitope (red) with N332 glycosylation (mannose indicated in green and GlcNAc in blue). (b) Multivalent analogue of the conjugate in panel a with presentation of three V3 glycopeptide epitopes. Reproduced from ref (199). Copyright 2018 American Chemical Society.

Minimal peptide sequences have been examined as epitopes of V3-glycan-specific bnAbs based on the HIV-1 glycopeptide immunogen.^198^ A vaccine was developed^198^ based on a synthetic three-component mixture containing a 33-mer V3 glycopeptide epitope with a high-mannose glycan at the N332 site (Figure 8), a universal T helper epitope P30, a 21-residue peptide derived from the tetanus toxoid,^198^ and lipopeptide Pam_3_CSK_4_. This self-adjuvanting system was shown to induce glycan-dependent antibody responses.^198^ This work was later extended to the synthesis of other analogous conjugates glycosylated at N332 as well as N301 and N295 sites in a study of glycan-reactive bnAb binding sites.^191^ Binding was studied via surface plasmon resonance and ELISA measurements. The same group later covalently linked the three components previously studied in mixtures to produce the conjugate shown in Figure 9, along with a multivalent version in which three copies of the N332 glycosylated V3 epitope are presented.^199^ Multivalent presentation significantly increased the immunogenicity of the V3 glycopeptide, and the antisera showed stronger binding to HIV glycoproteins than the monovalent glycopeptide.

Another HIV envelope glycoprotein that has been targeted is gp41.^188^ The HIV-1 fusion peptide, which comprises 15–20 hydrophobic N-terminal residues of the Env gp41 trimer subunit, is targeted by human bnAbs.^83^ The peptide includes the gp41_512–527_ sequence AVGIGAVFLGFLGAAG, regions of which were found (by molecular dynamics simulations) to be solvent-exposed. HIV envelope proteins such as gp120 and gp41 (and hemagglutinin from influenza virus membranes) undergo conformational changes that enable virus and host cell membranes to fuse.^200,201^ A shorter fusion peptide termed FP8 has the most prevalent sequence AVGIGAVF (residues 512–519).^202^ Immunization with this peptide followed by boosting with intact Env trimer can elicit therapeutically relevant cross-clade bnAbs in standard vaccine animal models. Neutralizing responses in mice can be generated by priming with FP8 linked to keyhole limpet hemocyanin (KLH) (see section 3.1) and boosting with prefusion-stabilized Env trimers.^202,203^ A SAPN coiled-coil construct has been created that incorporates the gp41 (HXB2 strain 662–682) membrane proximal external region (MPER) sequence at the N-terminus of the pentameric unit, which has been used to develop a potential adjuvant-free HIV-1 vaccine.^84^ The MPER epitope (LDKWASLWNWFNITNWLWYIR) was added so as to preserve the native α-helical presentation of the 4E10 gp41 antibody epitope. The peptide also incorporates a trimeric coiled-coil sequence and a sequence from *Plasmodium berghei* (see section 3.4). Activity against HIV-1 was assessed using rats after immunization with MPER-SAPNs. It was shown that MPER-specific antibodies were generated via the repetitive display of MPER antigen on the SAPN, although detectable neutralizing activity against HIV-1 was not observed in any of the sera.^84^ MPER is present at the C terminus of the exterior part of gp41 and is only partially accessible in the native Env spike.^85^ However, the flexible region is accessible for bnAbs during membrane fusion, that is, during the conformational transitions induced in the native Env spike upon binding to CD4 and co-receptors. A core gp41 MPER epitope ELLELDKW was identified by phage display,^86^ and later screening led to variant ELLELDKM, which shows better antibody binding properties.^85^ This peptide combined with a gp41 3S epitope to reduce CD4^+^ depletion, may be used to provide a dual-function vaccine to reduce and protect against infection while preserving CD4^+^ T cells.^85^

A (mouse model) HIV vaccine was developed that contains linked peptides representing an immune-dominant CTL epitope, P18, of gp160, located collinearly at the C-terminus of three cluster peptides.^204^ The former epitope is recognized by Th cells, of multiple MHC types from mice and humans. The dual-functional peptide exhibited both CD8^+^ CTL and CD4^+^ Th activity not observed when only P18 or cluster peptide mixtures were employed.^204^ HIV vaccines based on HIV-1 IIIB gp160 formulated with ISCOMS (immune-stimulating complexes) can generate CTLs that kill fibroblasts transfected with the gp160 IIIB gene, in response to the whole envelope protein or the immunodominant CTL epitope (RIQRGPGRAFVTIGK) of gp160.^87^

CTL epitopes from HIV glycoprotein (SLYNTVATL) or DNA polymerase Pol protein (ILKEPVHGV) or both have been linked to a Th sequence, specifically the promiscuous PADRE T-helper cell motif AKXVAAWTLKAAA (X = cyclohexylalanine) (see also section 2), fused to CpG-oligodeoxynucleotides as adjuvants (TLR9 agonists).^88^ In a mouse model of human HLA-A*02, the immunogenicity of linked DNA–peptide conjugates was enhanced compared to noncovalently linked mixtures of the same molecules, assessed by peptide-mediated cytotoxicity and IFN-γ release, and protection against viral infection is provided.^88^ The HIV-1 SLYNTVATL peptide has also been linked to an ionic complementary peptide EAK16-II (AEAEAKAKAEAEAKAK) that self-assembles into β-sheet fibrils, to potentially enhance immunogenicity.^89^ The conjugate peptide was studied in a mixture with TLR7/8 agonists resiquimod or imiquimod. DCs generated from HIV-positive patients exposed to the nanofiber formulation stimulated a significantly greater CTL response, compared to the DCs pulsed with the unconjugated peptide alone, or the unconjugated peptide mixed with TLR agonist.^89^

Adenovirus serotype 26 (Ad26) vectors have been exploited in the development of HIV vaccines that incorporate express mosaic HIV envelope (Env) and Gag-Pol immunogens [Gag = group antigens].^205^ This vaccine has proceeded to phase IIb clinical trials after earlier trials using rhesus monkeys.^205^ This adenovirus vaccine builds on the earlier MRKAd5 HIV-1 vaccine (Merck and Co., Inc.).^206,207^

A HIV vaccine strategy relying on the humoral immune system has been proposed that is based on a mixture of synthetic peptides comprising HIV-1 Env V3 sequences from HIV isolates, based on the conserved GPGR core sequence at the V3 tip region of gp120.^208^ Earlier, peptide-based ELISA was used to measure antibodies that specifically bind synthetic HIV fragment sequences (15-mers) derived from HIV serum specimens from Japanese patients with hemophilia A infected with HIV-1 subtype B.^209^ The GPGR tetrapeptide motif was present in 78% of strains.^209^ V3 peptides cyclized via disulfide bonds showed better HIV-1 neutralization behavior in rabbits compared to the linear homologous peptide.^90^ The constrained V3 peptides bearing the GPGR motif were linked to a 16-residue segment (KQIINMWQEVGKAMYA) of the gp120 C4 region, a known Th epitope. The constrained peptide also stimulated a significantly enhanced HIV-1 neutralizing response compared to that elicited by a gp120 construct with exposed V3 peptide.^90^

The HIV vaccine Vacc-4x is a candidate peptide-based HIV vaccine that has reached advanced clinical trials.^210−214^ It is a mixture of four modified peptides (20–27 residues) from p24 capsid^210^ that is designed to produce cell-mediated immune responses to HIV p24 Gag protein regions conserved between certain HIV strains.^212,214^

### 3.4 Malaria and Other Parasite Diseases

**Figure 10:**
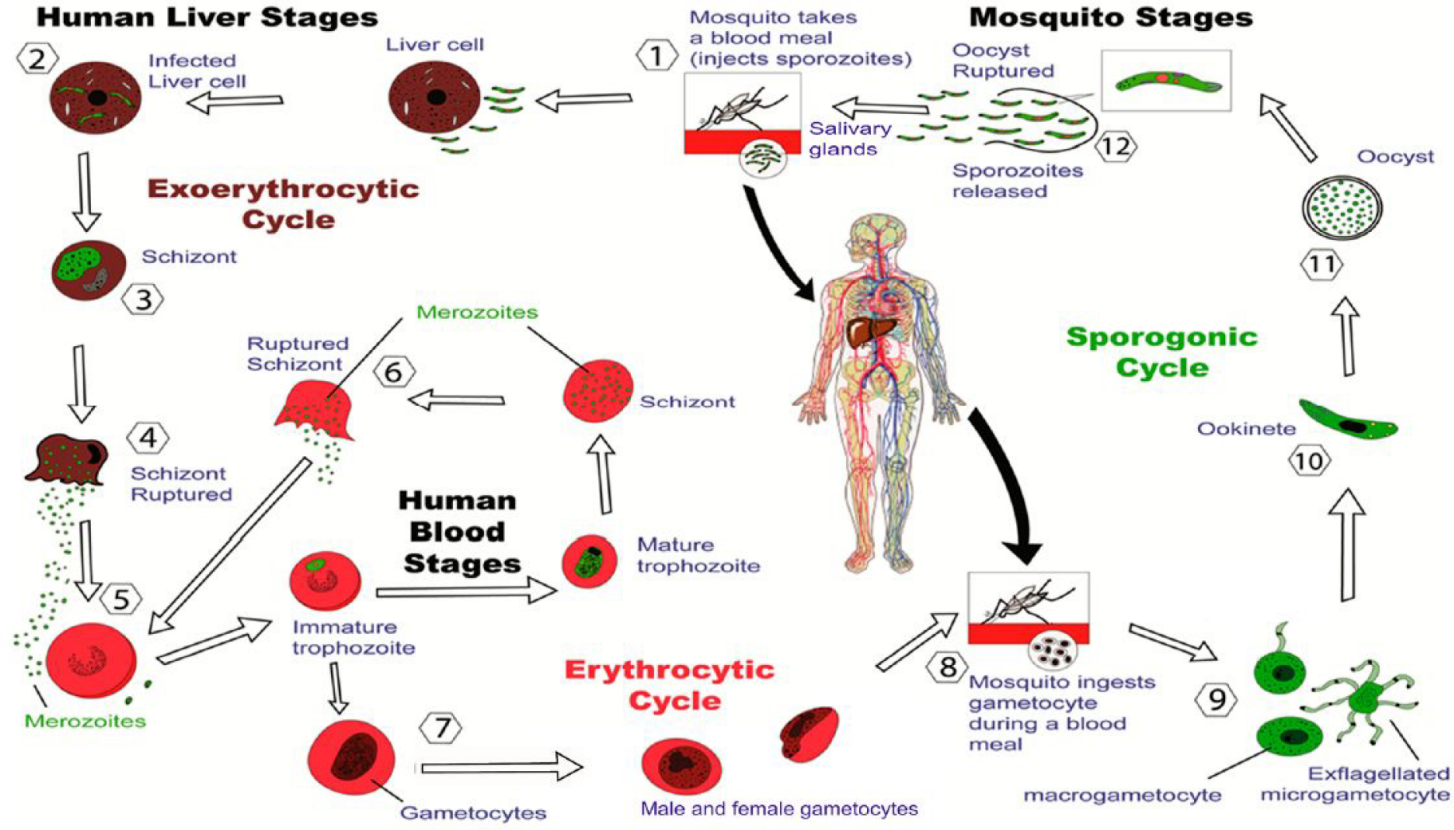
Stages of the malaria parasite life cycle. From ref (218).

Malaria is caused by sporozoites of parasites of *Plasmodium* species, especially *P. falciparum* and *P. vivax*. These are carried by *Anopheles* spp. mosquitoes and are transmitted when blood is ingested.^215,216^ It is estimated that around 200 million people per year contract malaria, and in 2019, about 400 000 people, 94% of whom were in Africa, died from the disease.^217^Figure 10 shows a schematic of the malaria parasite life cycle.^218^

Strategies have been suggested for anti-malaria peptide vaccines based on the *P. falciparum* life cycle (cf. Figure 10):^25,216,219^ (1) sporozoite spreading in the liver prevention (pre-erythocyte stage vaccines); (2) erythrocyte (red blood cell) entry inhibition (blood stage vaccines); and (3) induction of neutralizing antibody responses against the parasite’s gametocyte or ookinete stages in mosquitoes (transmission blocking vaccines). The most common malaria vaccines are subunit vaccines containing antigenic proteins or vaccines based on attenuated live parasite proteins.^25^ Subunit vaccines may be developed based on proteins with strong antigenic activity including the circumsporozoite (CS) protein and apical membrane antigen 1 (AMA-1). The CS protein is a ca. 42 kDa soluble protein that is needed for sporozoite development in the liver. Within the CS protein, a 37 tetrapeptide repeat, Asn-Pro-Asn-Ala (NPNA) (or equivalently NANP), along with a thrombospondin conserved domain, are essential immunogenic epitopes.^25,91,92^ The development of malaria subunit vaccines has recently been reviewed.^218^

**Figure 11:**
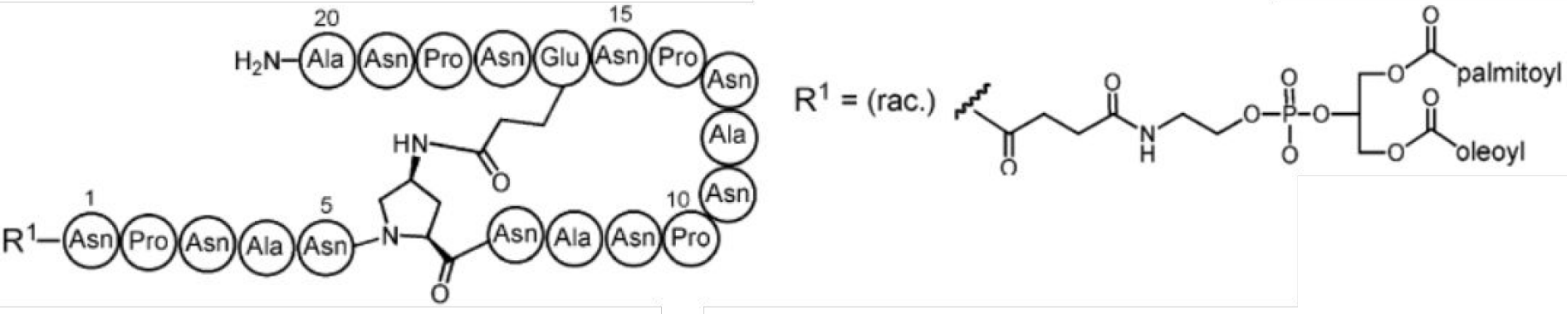
Peptide conjugate UK-39 developed as a malaria vaccine. Reprinted from ref (222), Copyright 2007, with permission from Elsevier.

The first approved malaria vaccine (RTS,S, trade name Mosquirix) is a recombinant subunit vaccine comprising a 188-residue truncated CS sequence expressed with a 226-residue hepatitis-B surface antigen (HBsAg) in yeast.^25,93,215^ This vaccine generated great excitement, following endorsement by the WHO in October 2021 of plans for widespread use in children. Vaccine RTS,S is generally delivered with the liposomal adjuvant AS01 containing QS-21 (a plant-derived saponin) and monophosphoryl lipid A (MPL).^220^ A review on RTS,S discusses other adjuvant formulations that have been investigated.^221^ The crystal structure of the ANPNA peptide (which contains the core CS tetrapeptide repeat mentioned above) has been determined.^93^ Antibodies against the NPNA sequence confer protection against malaria, and these have been analyzed. In fact, it was possible to obtain crystal structures of 1210 and 1450 antigen-binding fragment (Fab) with (NANP)_5_.^94^ These antibodies result from affinity maturation selection of B cells that express mutated antibody variants with improved antigen-binding properties. The understanding of the binding of the co-complex led to the development of UK-39 (Figure 11),^222^ a peptide–phosphatidylethanolamine (PE) conjugate containing a more stable cyclized structure of the loop containing two NPNA units.^25,223−226^ This peptide has been attached at the surface of immunopotentiating influenza virosomes (IRIVs) in clinical trial development; these are vesicles containing reconstituted influenza virus glycoproteins, which retain activity for binding to the cell surface and cell fusion and are used as antigen-delivery platforms that elicit B- and T-cell responses.^25,226−228^ Immunization of mice and rabbits with UK-39 at the surface of IRIVs elicited high titers of sporozoite cross-reactive immunoglobulins.^222^

A novel peptide-based malaria vaccine, R21, has recently progressed to phase 3 trials, following phase 2 trials that showed that 77% of the approximately 400 babies and infants in Burkina Faso in the trial were protected against disease after 1 year.^229^ Like RTS,S, R21 contains a peptide that is a fusion of CS and HBsAg sequences. However, R21 lacks the unfused excess HBsAg found in RTS,S and contains a different adjuvant, saponin-based Matrix-M (R21/MM).^230^ R21 has been shown to form virus-like globular particles by TEM imaging.^230^ Virus-like particles such as those formed from p33–HBsAg (p33 is a peptide derived from lymphocytic choriomeningitis virus) can actually stimulate APCs without adjuvant.^231^ A peptide fragment of p33, p33–41 (KAVYNFATM), shows a similar ability to form VLPs.^232^

Another candidate malaria vaccine was developed based on a peptide sequence from the C-terminal region (aa 282–383) of the CS protein of *P. falciparum*.^233^ A vaccine was formulated with Montanide adjuvant and in human trials was found to be well tolerated and able to produce a strong sporozoite-specific antibody response through CD4^+^ and CD8^+^ CTLs.^233^ A longer sequence, 181–276, from the C-terminal region of *P. falciparum* merozoite surface protein 3 (MSP3) has also been used in vaccine trials with Montanide or alum as adjuvant.^234^ Although vaccines with both adjuvants were immunogenic, that containing Montanide was found to give adverse reactions (inflammation).^234^ As the basis for malaria vaccine development, a series of α-helical peptides (30–70 residues) have been prepared and investigated based on screening of the *P. falciparum* 3D7 genome. This led to the identification of a series of coiled-coil domains of proteins thought to be present in the parasite erythrocyte stage.^235^ The array of synthesized peptides were all specifically recognized in immune sera of humans, though to different extents.^235^

CS-specific CTL have been generated by immunization by peptides from CS proteins from other malaria species, *P. berghei* and *P. yoelii*. The CS peptides correspond to a CTL epitope presented by MHC class l H-2K^d^ molecules or by Th cells.^236^ Use of both of both types of peptide prevented the induction of T cell tolerance and increased the magnitude of the CTL response.^236^

Another antigen that has been used in subunit vaccine development is the apical membrane antigen 1 (AMA-1), which is a type I integral membrane protein located at the merozoite surface (a merozoite is a cell produced by asexual reproduction that is released from red blood cells).^25^ AMA-1 is an 83 kDa integral membrane protein with sequence diversity (allelic variants), which is cleaved into a 66 kDa product upon merozoite release.^237^ The structure of AMA-1 has been examined and is found to be stabilized by multiple disulfide bonds.^238^ AMA-1 plays a central role in erythrocyte invasion by *Plasmodium* species.^25,239,240^ The critical residues involved in erythrocyte binding were identified and include the sequences DAEVAGTQYRLPSGKCPVFG, VVDNWEKVCPRKNLQNAKFG, WGEEKRASHTTPVLMEKPYY, and MIKSAFLPTGAFKADRYKSH. All conserved peptides were able to prevent merozoite penetration of red blood cells and merozoite development, indicating that these peptides are associated with *P. falciparum* invasion.^240^ AMA-1 has three subdomains in its ectodomain, and it appears that strain-specific epitopes in domain I are recognized by the majority of antibodies raised against the ectodomain.^241^ Since this domain shows considerable sequence variation in contrast to domain III (which contains more conserved epitopes), a virosomal formulation (IRIV) of a peptide that mimics the partly conserved loop I of domain III was developed that elicits parasite growth-inhibiting antibodies.^241^ A synthetic peptide comprising residues 446–490 of AMA-1 was attached at the N-terminus to a phosphatidylethanolamine lipid derivative (similar to the concept discussed above and conjugate structure shown in Figure 11), and the conjugate was incorporated into IRIVs as an antigen delivery system. Cyclized and linear versions of the peptide antigen both elicited antibodies that showed specific binding to parasite-expressed AMA-1, in a mouse model.^241^ Following encouraging animal study results with the conjugate containing a cyclized peptide (and the CS protein NPNA-conjugates discussed above), human clinical trials have been conducted.^25^

Collier’s group has also used the Q11 peptide discussed in section 2 as a scaffold for the malaria peptide antigen (NANP)_3_ from the CS protein of the *P. falciparum* protozoan parasite.^242^ The conjugate retains a β-sheet fibril structure and was found to be effective in raising antibodies, the response lasting up to 40 weeks. Antibody production was shown to be T cell- and MyD88-dependent (studied using MyD88 knockout mice; the MyD88 protein plays an essential role in immune cell activation through TLRs) whereas antibody production was not abolished in knockout mice lacking functional TLR-2, TLR-5, or NALP3 (also known as NLRP3, a pattern recognition receptor protein involved in the inflammasome pathway). The (NANP)_3_–Q11 conjugate could be co-assembled with OVA–Q11 without diminishing the immunogenicity of either on its own.^242^

**Figure 12:**
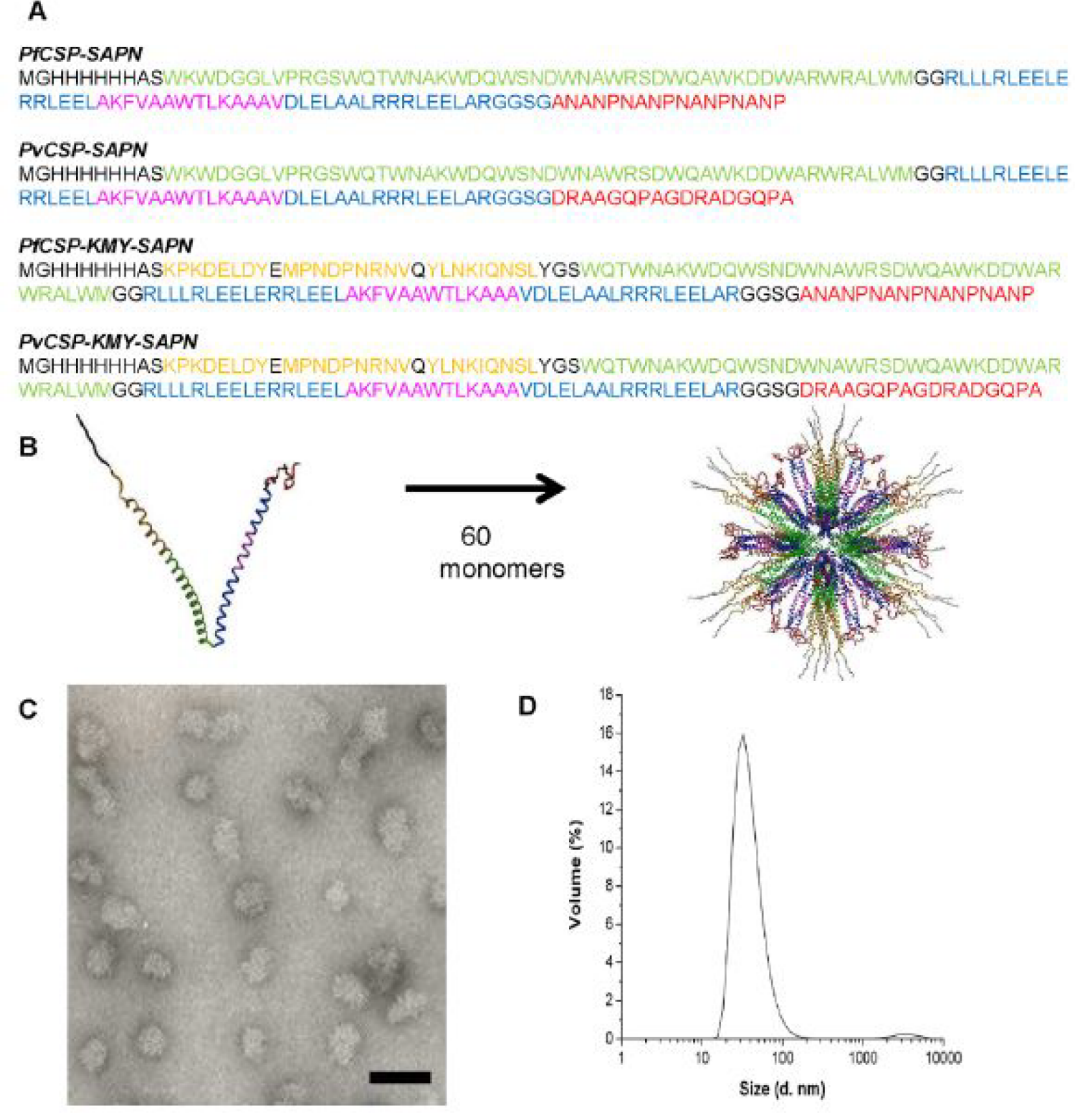
Self-assembling peptide nanoparticles (SAPNs) for anti-malaria vaccination.^243^ (a) Peptide sequences. Color coding as follows: black, flanking regions (thrombin cleavage site, His-tag, proteosome cleavage sites and linkers); red, B cell epitopes predicted for *P. falciparum* (NANP repeats, see also ref (242) and associated discussion within the text) or *P. vivax* CS protein repeat region (DRAAGQPAGDRADGQPA); green, coiled-coil pentamer domain; blue, coiled-coil trimer domain; yellow, predicted human HLA-restricted CD8^+^ T cell epitopes from *P. falciparum* CS protein; magenta: universal CD4 T-helper epitope (PADRE) within the trimer domain. (b) Schematic of packing of coiled-coils into spherical nanoparticles. (c) TEM image of nanoparticles (scale bar = 100 nm). (d) Size distribution of the nanoparticles from dynamic light scattering. From ref (243).

Spherical particles with a diameter of about 40 nm formed by the self-assembly of ∼125-residue coiled-coil peptides (miniproteins) were used as vaccine nanoparticles for the malaria parasite *P. falciparum* CS.^243^ The peptides contain sequences designed to form coiled-coil pentamers or trimers with several functional epitopes (Figure 12a,b). Figure12c shows an electron micrograph image, revealing spherical nanoparticles, along with a schematic of the modeled packing of approximately 60 peptide chains into such a structure (Figure 12b). These SAPNs raise long-lasting antibodies in mice and long-lived CD8^+^ T cells. The latter was achieved by incorporating KMY CD8^+^ T cell epitopes into the nanoparticles (Figure 12), where KMY refers to **K**PKDELDY, **M**PNDPNRNV, and **Y**LNKQNSL.^243^ In a related work, a B cell immunodominant repeat sequence (DPPPPNPN)_2_D from the malaria parasite *P. berghei* CS protein was similarly displayed on coiled-coil peptides of the same design.^244^ The non-adjuvanted vaccine was shown to provide extended protection against malaria in rodents.^244^ Vaccines for toxoplasma in mice were also developed using related coiled-coil oligomerization domains in a single linear peptide.^245^ The pentameric and trimeric coiled-coil domains were linked via spacers (cf. Figure 12) to the GRA_720–728_ (LPQFATAAT) peptide and a PADRE-derived CD4 helper epitope (ERFVAAWTLRVRA) within the same peptide sequence. This GRA peptide is based on GRA7, a potent antigen that elicits IFN-γ from CD8^+^ T cells and is expressed in *Toxoplasma gondii* infections. Similar to the previously discussed SAPNs, these peptides self-assemble into icosahedral nanoparticles, as imaged by TEM, with a diameter ∼38 nm determined by dynamic light scattering (cf. Figure 12d).^245^

### 3.5 SARS-CoV-2 and Related Coronaviruses

**Figure 13:**
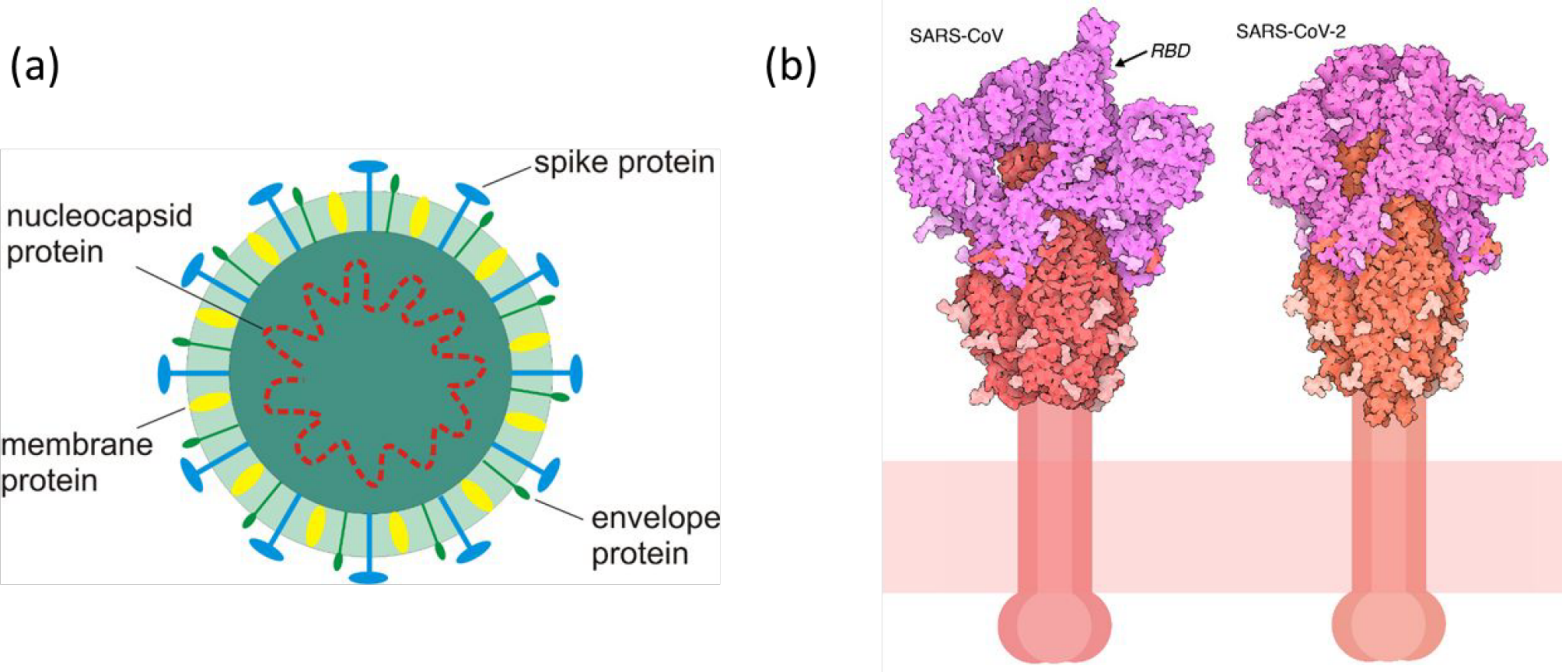
(a) Representative schematic structure of a coronavirus such as SARS-CoV-2. (b) (left) Spike protein from SARS-CoV with one receptor binding domain (RBD) raised and (right) a closed conformation of the SARS-CoV-2 spike protein. The S1 fragment is colored magenta, and the S2 fragment is red, with glycosylation in lighter shades. From https://pdb101.rcsb.org/motm/246.

**Figure 14:**
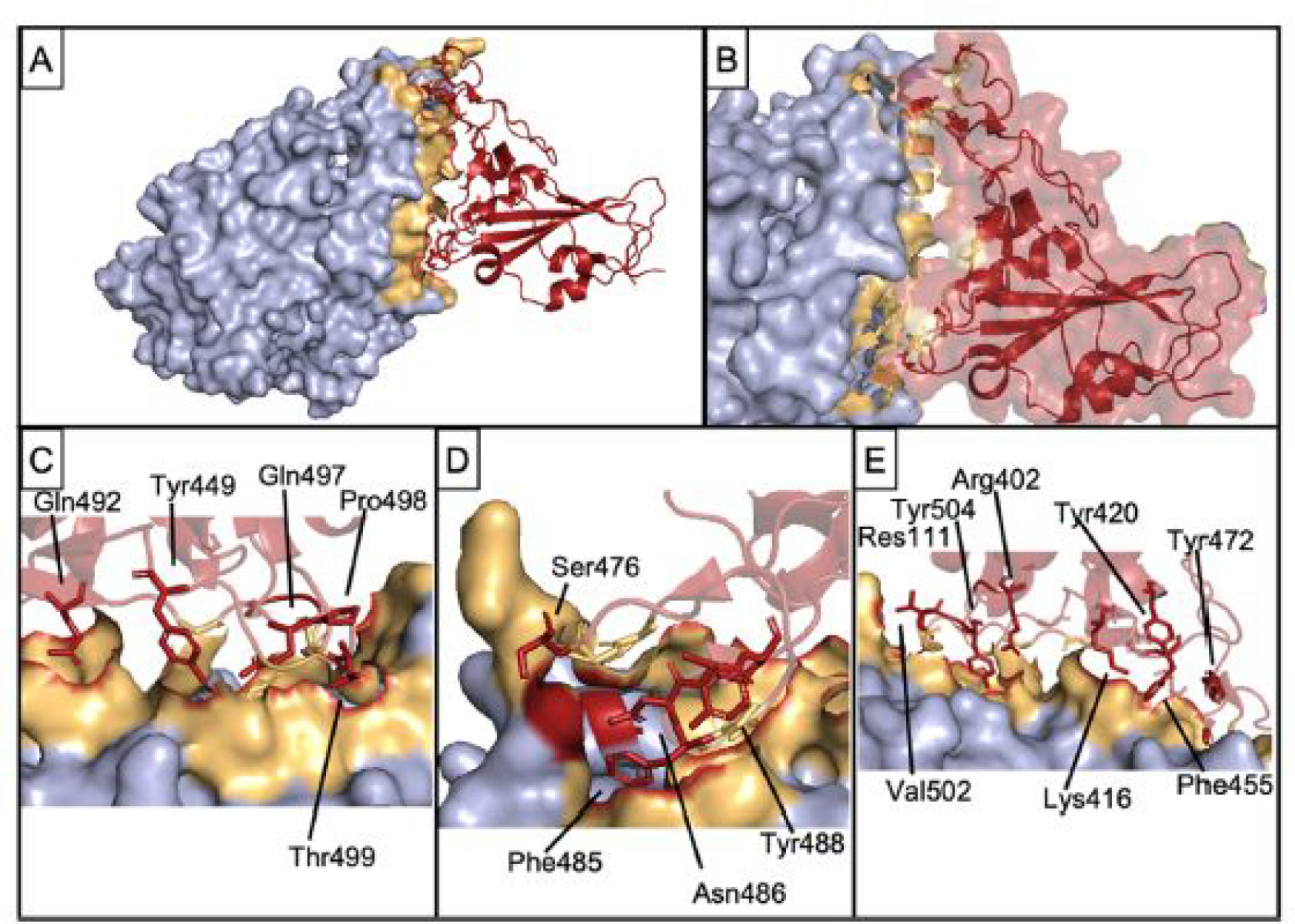
Interaction of ACE-2 (light blue and yellow surfaces) with spike RBD (red): (A) bound complex; (B) close-up of the interaction interface; (C–E) Highlighting important residues from spike RBD involved in complex formation. PDB 7DMU. Reproduced with permission from ref (250). Copyright 2021 Wiley-VCH.

The global COVID-19 pandemic caused by the SARS-CoV-2 [SARS, severe acute respiratory syndrome] virus stimulated the incredibly rapid development successful vaccines based on mRNA and adenovirus vectors, and others.^1−6,246−249^ To date, peptide–epitope based vaccines for SARS-CoV-2 have not reached practice, although a number of trials have been launched (see, for example, Table 1). The structure of a coronavirus is illustrated in Figure 13a, and the spike protein structures are highlighted in Figure 13b. Key to recognition of human cells are the spike (glyco)proteins, which interact with the ACE2 cell receptor (Figure 13b). SARS-CoV-2 proteins have now been sequenced, and it has been possible to identify key regions involved in the binding of the spike protein to target cells, and these represent potential targets for therapeutic interventions. A review is available that focuses on peptide therapeutics for SARS-CoV and SARS-CoV-2 and contains, among other valuable information, a table of peptide inhibitors that have been identified from *in vitro* and *in silico* approaches that target interactions mediated by the spike receptor binding domain (RBD).^250^ Other strategies are discussed including peptide inhibition to target the ACE-2 receptor itself, fusion inhibition by targeting heptad repeat domains HR1 and HR2 and inhibition of binding between the ACE-2 receptor and the RBD (Figure 14). An early review on SARS-CoV-2 modeling activities introduces several of the widely used immunoinformatics methods (including many discussed below) as well as summarizing research done early in the pandemic that identified T cell peptide epitopes and studied their HLA-binding activities.^251^ A review of angiotensin receptor blockers as potential targets for SARS-CoV-2 treatments highlights that viral peptides may not be effective against future coronavirus outbreaks, as mutations could render them inactive.^252^ Reviews on SARS-CoV-2 vaccines under development includes discussion of several vaccine candidates based on peptide epitopes.^247−249,253,254^ It should be noted that this is a very fast moving field and these reviews are often rapidly outdated by fast emerging knowledge and technologies. There is now a very extensive literature on SARS-CoV-2 including identification of many peptides as T cell epitopes as well as other studies on viral protein/cell receptor binding inhibition. The following is a selection of key reports to date, including examples of both experimental and computational studies. A few examples are also discussed of earlier work on related coronaviruses MERS-CoV [MERS: Middle East respiratory syndrome] and SARS-CoV (from the 2002–2004 SARS outbreak).

Peptide epitopes have been identified from COVID-19 patient screens using the VirScan phage-display platform which uses an oligonucleotide library encoding 56-residue peptides tiling every 28 amino acids (and 20-mers spanning every 5 aa) across the proteome.^255^ Among the peptides highlighted, ten epitopes were thought likely to be recognized by neutralizing antibodies. The authors highlighted the relevance of such findings to the development of diagnostics and the isolation of antibodies including potential neutralizing antibodies.^255^ In another study of sera from COVID-19 patients, a library of B cell peptides was produced that spans the whole S glycoprotein of SARS-CoV-2 (or SARS-CoV) in series of five overlapping peptides.^256^ This led to the identification of two dominant immunoglobulin regions on the SARS-CoV-2 spike glycoprotein recognized by sera from patients recovering from COVID-19, one close to the RBD.^256^ Peptide epitope targets for T cell recognition have been selected based on predictions of SARS-CoV-2 HLA-binding peptides using the SYFPEITHI database and NetMHCpan artificial neural network server. T cells amplified *in vitro* from patients exposed to SARS-CoV-2 (or controls) enabled the identification of a series of HLA-binding peptides that are natural T cell epitopes.^257^ The peptides specific for SARS-CoV-2 enabled post-infection T cell immunity to be detected, even in seronegative convalescent patients, which thus established similarity to common cold coronaviruses. Pre-existing T cell responses in were observed for 81% of unexposed individuals.^257^ On the other hand, Ferretti et al. reported SARS-CoV-2 epitopes that are widely shared by CD8^+^ T cells of COVID-19 patients but show low cross-reactivity with other seasonal coronaviruses.^258^ All memory CD8^+^ T cells for a particular COVID-19 patient were screened, for every HLA allele, against every epitope in the SARS-CoV-2 virus and the four seasonal coronaviruses responsible for the common cold. CD8^+^ T cells were cocultured with an array of engineered target cells that express one HLA allele, each of the target cells expressing a unique 61-residue SARS-CoV-2 protein fragment. These sequences were found to be processed naturally by the target cells, the appropriate peptide epitopes being displayed on MHC class I molecules. The authors also found that most epitopes are not within spike protein sequences.^258^ A SARS-CoV-2 peptide microarray was constructed using 15-mer peptides (with a 5 residue overlap across the proteome) to analyze antibody binding.^259^ In addition, the utility of SARS-CoV-1 antibodies in the detection of the SARS-CoV-2 nucleocapsid protein was demonstrated. The authors also identified B cell epitopes for SARS-CoV-2 antibodies in the serum of ten COVID-19 patients.^259^ Two HLA-A*02:01-restricted CD8^+^ T cell epitopes specific for SARS-CoV-2 have been identified: A2/S_269–277_ (YLQPRTFLL) and A2/Orf1ab_3183–3191_ (FLLNKEMYL).^95^ T cells corresponding to the former epitope are detected at comparable frequencies in acute and recovering patients (and at levels above those for uninfected donors) though with a weaker response than for influenza or Epstein-Barr virus A2 sequences. The former epitope shows high conservancy with MERS-CoV and SARS-CoV-1, and the latter shows 100% conservancy with SARS-CoV-1.^95^

Significant cellular responses have been observed using splenocytes of mice given a SARS-CoV-2 spike protein RNA/lipid nanoparticle vaccine candidate.^260^ In particular IFN-γ was produced, upon re-stimulation with SARS-CoV-2 peptides from a pool of 15-mers.^260^

Extensive computational modeling has been undertaken to examine potentially useful epitopes. In one example, a computational screening study of SARS-CoV-2 proteins including nucleocapsid proteins, membrane glycoproteins and surface spike glycoproteins has identified epitopes for B cells, Th cells, and CTLs.^261^ The computational data suggest that the epitopes can be used in a vaccine that is non-toxic, non-allergenic, and able to elicit cell- and humoral-mediated immune response. Lin et al. used immune informatics methods to identify B and T cell epitopes for the membrane glycoprotein (M), surface glycoprotein (S), and nucleocapsid protein (N) of SARS-CoV-2 and evaluated their antigenicity and interactions with HLA alleles.^262^ Analysis of toxicity, allergenicity, physiochemical properties, and stability confirmed the selectivity and specificity of the candidate epitopes.^262^ An immune informatics approach has been adopted using the viral genome to identify highly immunogenic B cell epitopes and nearly 500 HLA-restricted T cell epitopes.^96^ A total of 30 peptides were selected as potential vaccine candidates, 26 of them derived from the SARS-CoV-2 spike protein, 2 from the membrane protein, and 2 from the envelope protein. A docking study revealed that sequences FIAGLIAIV and FVSEETGTL strongly bind different types of HLA. A selection of these were used in the development of peptide vaccines which elicited cellular and humoral responses specific to antigens in mice.^96^ Screening of the immune epitope database (IEDB)^50,51^ revealed a series of B cell peptide epitopes and ProPred-I and ProPred servers were used to select MHC-I and MHC-II binding T cell epitopes respectively within pre-identified B cell epitope regions.^263^ The VaxiJen server^264^ was then used to assess potential antigenicity. The 13 MHC-I and 3 MHC-II antigenic epitopes identified were connected using (EAAAK)_3_ linker peptides to construct a model peptide vaccine, the docking of which to TLR-5 was modeled.^263^ In a separate study, the IEDB was also used to identify T cell epitopes and to model MHC class I and class II binding along with *in silico* docking analysis and VaxiGen server antigenicity testing.^97^ Lead candidate peptides identified were YVYSRVKNL, SLVKPSFYV, and LAILTALRL, and these dock well with HLA-A*02:01.^97^ An *in silico* analysis has been performed of binding affinity of viral peptide and MHC class I for HLA-A, -B, and -C genotypes for all 8-mer and 12-mer SARS-CoV-2 peptides (48 395 unique peptides).^265^ HLA-B*46:01 contained the fewest predicted binding peptides for SARS-CoV-2, indicating that with a person with this allele may be particularly susceptible to COVID-19,^265^ as for SARS-CoV.^266^ On the other hand, HLA-B*15:03 was presented by highly conserved SARS-CoV-2 peptides common to many human coronaviruses to the greatest extent, suggesting that it could provide cross-protective T cell-based immunity.^265^

Another group used the IEDB to identify T cell epitopes in SARS-CoV2 and SARS-CoV, which have high sequence homology.^267^ Using these predicted pools of epitopes, SARS-CoV-2 CD4^+^ and CD8^+^ T cell responses following infection in recovered COVID-19 patients were analyzed.^268^ SARS-CoV-2 T cell responses cross-reactive with those from other common coronaviruses were observed in healthy donors, indicating the possibility of pre-existing immunity in the human population.^268^ Another study also examined the immunodominant memory T cell responses specific to SARS-CoV-2 in patients who recovered from COVID-19.^269^ This was evaluated *in vitro* using peptides covering the full proteome of SARS-CoV-2. The extent and magnitude of T cell responses were notably enhanced in severe cases. T cell responses specific to the spike or the total response were found to correlate with spike-specific antibody production. The authors identified a series of 41 peptides with CD4^+^ or CD8^+^ epitopes, including six immunodominant epitope groups.^269^ Immunoinformatics methods (NetCTL, CTLPred, BepiPred, etc.) have been employed to find CTL and B cell epitopes within the SARS-CoV-2 surface glycoprotein sequence, and binding of CTLs with MHC-1 was analyzed.^270^ Similar techniques were used by Crooke et al., who identified 41 T cell epitopes and 6 B cell epitopes as potential targets for the development of peptide-based vaccines.^271^ HLA-binding sequences (9-mer peptides) were also screened using NetMHC and NetCTL to analyze proteasome cleavage and transport.^272^ Potential CD8^+^ T cell cross-reactivity conferred by other coronavirus strains against SARS-CoV-2 has also been examined using *in silico* mapping (using IEDB, NetMHC, and other tools) of CD8^+^ T cell epitopes shared between coronaviruses.^273^ This follows an examination of the immunogenicity of SARS-CoV-2 sequences (and correlation to related sequences in the IEDB) as well as identification of novel HLA-binding and TCR-recognition sequences by the same group.^274^ Similar informatics methods (NetMHC, VaxiJen, AntigenPro, ToxinPred, and AllerTop servers) were used by Samad et al. to identify B and T cell epitopes and to predict their immunogenicity.^275^ The binding interaction between the vaccine and a toll-like receptor (TLR4) was also probed.^275^ Related tools were used in a search for immune cell epitopes in reports from other groups.^98,276^ Rakib et al. also modeled HLA-B*15:01 binding and identified lead candidate peptides WTAGAAAYY and GAAAYYVGY.^98^ The IEDB and VaxiJen servers were used in a similar study identifying B and T cell epitopes and examining docking interactions with TLR3.^277^ Inspired by the fact that the SARS-CoV-2 spike protein interacts directly with the peptidase domain (PD) of the ACE2 receptor, the interaction of a PD peptide sequence that was shown to block interaction with the spike protein has been modeled using molecular dynamics simulations, thus providing a possible candidate inhibitor peptide.^278^

The sequence RSAIEDLLFDKV occurs in many coronavirus spike proteins such as those from the human common cold coronavirus or various animal coronaviruses.^99^ It is located immediately following the second (S2′) cleavage site of SARS-CoV and MERS-CoV,^279^ and closely related sequences have been identified in SARS-CoV-2.^99^ Among related sequences, KRSFIEDLLFNKV is a well-conserved epitope located near one of the established cleavage sites of SARS viruses that appear to be necessary for virus activation during cell entry.^100,101^ This sequence was identified from bioinformatics, from the sequence of the spike protein obtained from extracted nucleic acid or protein and prediction and alignment of surface sites and subsequent investigation of binding to targets.^100^ Antibody epitopes of spike proteins were analyzed using the BepiPred-2.0 bioinformatic tool, making a comparison between MERS-CoV, SARS-CoV, and SARS-CoV-2, and unique epitopes, shared epitopes, and epitopes shared by multiple antigens (public epitopes) were identified.^102^ Two high-score epitopes located in the RBD (peptides ASTEK and PKKS) with the potential to block the interaction between ACE2 and the spike protein to inhibit SARS-CoV-2 infection were identified.^102^

Potential epitopes derived from the SARS-CoV-2 sequences for HLAs that are often present in Japanese people have been subjected to bioinformatics screening, enabling the identification of a large number of peptide epitopes likely to have high affinity to HLA class I and II molecules, respectively, potentially able to elicit T cell responses.^280^ Binding affinity was assessed using the NetMHC family of software.^280^ A variety of bioinformatics tools were used to predict the binding affinity between 15-mer and 9-mer peptides from the possible space of SARS-CoV-2 peptides (the peptidome) with large numbers of MHC class I and HLA alleles.^281^ A considerable number of peptide–HLA complexes (pMHCs) were identified with a predicted binding affinity less than 500 nM.^281^

As an alternative to identification of immunogenic peptide epitopes, *in silico* methods have been used to screen for protease inhibitors. A database docking screen was followed by molecular dynamics (MD) simulations of the docking of four lead candidate peptide and peptide-like small molecules into the protease binding site.^282^

Peptides associated with the Th1 adaptive immune response, specifically antiviral cytokines from interferon gamma (IFNγ) core sequences have been shown to have cell nuclear localization properties similar to that of the much longer parent protein. In particular, heptapeptide RKRKRSRC has cell nucleus localization behavior,^283^ the sequence of this peptide being similar to the SV40 nuclear localization sequence (NLS) PKKKRKV in the SV40 (simian vacuolating virus 40) large T-antigen NLS.^284^ This peptide and related antiviral cytokines including TNFα and interleukin-12 are virus-specific effectors for T cell antigens involved in the immune response to coronaviruses.^285^

*In silico* methods have also been employed to identify peptide epitopes for MERS-CoV, leading to potential vaccines in preclinical development.^286,287^ Computational analysis techniques including use of several tools within IEDB were employed to identify T and B cell epitopes for potential use in a multiepitope vaccine, and interactions with HLA alleles and TLR-3 were also modeled.^288^ Immunoinformatics and computational methods (using IEDB, BCPRED, and Vaxijen servers among others) have been used to identify highly conserved B and T cell epitopes for the MERS-CoV spike protein, and their antigenicity and interactions with the HLA B7 allele were evaluated.^103^ Among B cell epitopes, the highest antigenicity was found for QLQMGFGITVQYGT, which was also highly immunogenic. Considering T cell epitopes, MHC class I peptide YKLQPLTFL and MHC class II peptide YCILEPRSG were found to be highly antigenic.^103^ An immunoinformatics-based genome-wide screen of vaccine targets showed that the MERS-CoV nucleocapsid protein is a better protective immunogen compared to the S protein, with high conservancy and potential to elicit both neutralizing antibodies and T cell responses.^289^ In addition, B cell, Th, and CTL epitopes were screened leading to multiple identified sequences.^289^ Software from the IEDB and other resources has also been used to predict MERS-CoV epitope vaccines based on sequences from the S glycoprotein or the envelope protein (or modified sequences).^290^ Such sequences can elicit both neutralizing antibodies and responses from B cells, Th cells, and CTLs.^290^ Heptad repeat 1 (HR1) peptide inhibitors have been designed to disrupt membrane fusion mediated by HR1/HR2 between MERS-CoV and host cells.^291^ In particular, a 42-residue α-helical peptide exhibits potent inhibitory activity that can be further enhanced in a peptide–gold nanorod complex.^291^

Computational methods were used to identify epitopes for vaccine development for SARS-CoV following the first SARS outbreak in 2003. Bioinformatic analysis led to the prediction of two epitopes (N1 and N2) from the nucleocapsid protein, which were then studied experimentally.^292^ Antibodies induced by these peptides had a high binding affinity to the nucleocapsid protein of SARS-CoV and N1 peptide-specific IgG antibodies were detectable in the sera of SARS patients after immunization.^292^ A software-based procedure was used to identify T cell epitopes in the SARS-CoV S protein.^293^ The immunogenicity of HLA-A*02-restricted T cell epitopes was investigated in patients who had fully recovered from SARS-CoV infection, and a specific T cell response was indeed elicited.^293^ Five immunodominant sites have been identified on the SARS spike protein via Pepscan analysis using a set of synthetic sequences spanning the entire S protein sequence using SARS patient sera and antisera from small animals immunized with inactivated SARS-CoV.^294^ It was found that site IV situated in the middle of the S protein sequence (residues 528–635) is an important epitope, a fragment of which, S_603–634_, reacted with all the convalescent SARS patient sera, indicating its potential application as an antigen.^294^ A virus-like particle has been developed as a SARS-CoV immunogen.^104^ The VLP comprises a designed coiled-coil peptide that contains a pentameric sequence, a trimeric domain, and the SARS-CoV spike protein C-terminal heptad repeat region (HRC) peptide SVVNIQKEIDRLNEVAKNLN, which is a B cell epitope. The peptide aggregates into nanoparticles (predicted to be polyhedra) with a diameter around 25 nm, and the nanoparticles elicit antibodies in BALB/c mice and demonstrate neutralization activity *in vitro*.^104^ A review on vaccine candidates for SARS-CoV is available that focuses on the spike protein as target.^295^

### 3.6 Other Infections and Conditions

A peptide vaccine for lymphocytic choriomeningitis virus (LCMV) was examined.^105^ The 15-mer peptide RPQASGVYMGNLTAQ, which is a T cell epitope of LCMV nucleoprotein, stimulated a specific CTL response in mice. The same peptide formulated with incomplete Freund’s adjuvant was shown to protect mice against subsequent infection with live virus.^106^

The Q11 peptide discussed in section 2 was used as a scaffold to co-assemble T and B cell epitopes for vaccine development.^73^ Peptide Q11 was coupled to either a CD4^+^ T cell epitope (PADRE, aKXVAAWTLKAa, X= cyclohexylalanine, a = d-alanine) or a B cell epitope (E214, FEGTEDAVETIIQAIEA) from *Staphylococcus aureus* via an SGSG linker. Fibrils from the co-assembled peptides were imaged by TEM. Peptide PADRE–Q11 elicited a T cell response (in contrast to the PADRE peptide itself), whereas E214–Q11 did not raise antibodies in the mouse model but the co-assembled E214–Q11/PADRE–Q11 did. The authors note that optimization of T follicular helper (Tfh) response is important for human antibody response, especially for vaccines against bacterial infection or influenza.^73^

A peptide from murine respirovirus (formerly Sendai virus) that is recognized by CTLs was identified using recombinant virus constructs containing separate genes of Sendai virus, in particular a series of peptides that span a nucleoprotein gene product.^107^ Mice immunized with the peptide HGEFAPGNYPALWSYA (positions 321–336 of the virus NP) were protected against a lethal virus dose. Shorter Sendai virus epitopes (down to 9 residue FAPGNYPAL) also stimulate immunogenicity via CTLs without assistance from Th cells.^108−110^ The latter study also shows loading of MHC class I using an adenovirus type 5 E1A protein sequence CDSGPSNTPPEIHPVV via H-2D^b^ binding.^110^

The specific features of receptors of CTLs that recognize an antigenic peptide associated with vesicular stomatitis virus (VSV) presented by the class I MHC molecule H-2K^b^ have been examined, based on peptide RGYVYQGL, which comprises residues 52–59 of the nucleoprotein.^111,112^ Even single amino acid substitutions influenced recognition by TCRs in a transgenic mouse model.^112^

T cell epitopes of human glutamic acid decarboxylase GAD65 protein are associated with insulin-dependent diabetes mellitus (IDDM).^113^ CTL clones specific to GAD65 antigens were isolated from two patients with congenital rubella syndrome (CRS)-associated IDDM. Overlapping T cell epitopes (9-mer peptides) recognized by both CD4^+^ and CD8^+^ CTL clones were identified as sequences bounded by GAD65 255–266, RFKMFPEVKEKGMAG, or GAD65 276–285, FTSEHSHFSL, respectively.^113^

A potential B cell epitope-based vaccine termed BM32 as been developed for immunotherapy of grass pollen allergy and is based on recombinant proteins incorporating a series of grass pollen antigen peptides with hepatitis B surface protein domain as an immunoactive carrier.^296^ The vaccine was found to be well tolerated and able to reduce allergic reactions as well as allergen-specific T-cell responses via IgG antibodies.^296^ Immunotherapy with BM32 can also induce antibodies protecting against hepatitis B infection.^183^

## 4 Cancer Immunotherapy Peptides

The manipulation of the immune response to treat cancer in immuno-oncology is attracting great interest, with the aim to circumvent the immunosuppressive evasion mechanisms used by cancer cells. The main focus has been to tune the responses of T cells, since these cells are able to clear tumors. Cancer cells are characterized by overexpression or mutation of certain proteins compared to normal healthy cells. Thus, proteins (or genes) that are expressed differently in cancer cells than in healthy cells or in a mutated form are targets for immunotherapies. The following discussion covers a number of tumor-associated antigens, including mucin 1 (MUC1), human epidermal growth factor receptor 2 (HER-2), cancer–testis antigen 1 (NY-ESO-1), melanoma antigen recognized by T cells 1 (Melan-A or MART-1), prostate-specific antigen (PSA), and others. Antigens such as glycoprotein 100 (gp100), prostatic acid phosphatase (PAP), and melanoma antigen-encoding gene (MAGE) are discussed elsewhere, along with neoantigens that result from tumor-specific mutations.^297^ Tumor neoantigens are the subject of focused reviews.^298−300^ Peptide-based vaccines for cancer have also been reviewed elsewhere.^300−304^ In addition, the uses of peptides to target cancers caused by infective agents, for example, cervical cancer caused by human papillomavirus (HPV), are discussed.

### 4.1 HER-2

HER-2 (also known as HER-2/neu) is a protein within the human epidermal growth factor receptor family, and over-expression of this oncogene is involved in the development and progression of some aggressive breast cancers. It is also associated with certain ovarian, stomach, lung, and uterine cancers. The HER-2 signaling pathway is involved in cell growth and division, and over-expression and gene amplification of HER-2 are linked to tumor cell proliferation and anti-apoptotic signaling, as observed for 15–30% of human breast cancers.^25,305,306^ Monoclonal antibody therapies including trastuzumab (Herceptin) and pertuzumab that target HER-2 are already used as passive immunization breast cancer drugs; however, immunotherapies based on active peptide epitopes are attracting considerable interest.^119^ Synthetic peptide sequences from HER-2 have been developed as minimal epitopes recognized by ovarian tumor-reactive CTL. Based on this, the nonapeptide E75 (HER-2_369–377_, KIFGSLAFL) was identified, and it was shown that this can be recognized specifically by CTL on ovarian tumors.^114^ A number of other HER-2 peptides were preferentially recognized by one or two CTL cell lines, indicating that both common and specific HER-2 epitopes may show immunoactivity against ovarian tumors. Another group reported that these peptides can be processed naturally as a gastric cancer tumor-associated antigens recognized by CTLs that are tumor-specific and HLA-A*02-restricted.^307,308^ The relative binding affinity of nona- and deca- peptides derived from HER-2 to HLA-A*02.1 was also determined by this group.^307^ Tumor-associated lymphocytes isolated from patients with cancers of the breast or ovary, enabled the identification of several such peptides.^309^ These tumor-associated CTLs are also able to lyse other tumors, including those from non-small-cell lung, colon carcinoma, renal cell carcinoma, and pancreatic cancers, indicating that HER-2/neu epitopes are common to various types of epithelial tumors.^309^ Peptide E75 combined with GM-CSF (granulocyte–macrophage colony-stimulating factor) was used in a vaccine (nelipepimut-S, also known as NeuVax) progressed to phase III clinical trials as a breast cancer treatment.^118,310−312^

Another HER-2 fragment-based peptide developed as a breast cancer immunotherapy is GP2, a fragment of the transmembrane portion of the HER-2/neu protein (654–662, IISAVVGIL).^116,117^ This was the subject of phase and I and II trials in combination with GM-CSF.^116,117,119^ This trial showed that the GP2 vaccine has a good safety profile and suggests that the clinical utility of vaccination, particularly for patients with HER2 overexpression who received the full vaccine series.^117^ A further peptide based on a HER-2 sequence that has been used in a breast cancer vaccine trial (again with GM-CSF) is based on AE-36, with sequence GVGSPYSRLLGICL (HER-2/neu 776–790).^118,119^ The addition of an N-terminal four-amino-acid LRMK sequence to give AE-37 was found in a phase I clinical trial to increase vaccine potency when compared with the unmodified peptide epitope.^118^

A HER-2 peptide-based vaccine was designed based on computer modeling of antibody binding regions of HER-2.^120^ Seven B cell epitopes from HER-2/neu were prepared and linked to a tetanus toxoid sequence and used for immunization in BALB/c mice. Immunizations with peptides P4 (PESFDGDPASNTAPLQPEQLQ) or P7 (YMPIWKFPDEEGAC) or a combination of P7 with P6 (CRVLQGLPREYVNARHC) induced anti-peptide antibodies.^120^ Since it is known that immune response polarization towards the Th1 path (Figure 1) is important for tumor prevention, the addition of the adjuvant IL-12 to a mixture of P4, P6, and P7 (again linked to tetanus toxoid through C-terminal cysteine residues) was investigated, with the aim to increase the potency of the breast cancer vaccine.^313^ These peptides were used in a subsequent phase I trial using virosomes (IRIVs) incorporating the three peptides.^314^ The authors report that this multipeptide vaccine is well tolerated, safe, and effective in overcoming immunological tolerance to HER-2/neu. Following these trials, further improvement of immunogenicity was achieved by linearly linking the peptides to give P467, and this peptide was coupled either to virosomes or to diphtheria toxoid CRM197, which, along with adjuvant, was used as a metastatic breast cancer vaccine.^315^ This multiepitope vaccine induced polyclonal antibodies with anti-proliferative activity against HER-2/neu, and on the basis of these promising findings, phase II trials were launched. This technology has been taken forward by Imugene as HER-Vaxx (IMU-131), a treatment for metastatic gastric cancer.^316^

Distinct HER-2 B cell epitopes have been used as the basis of vaccines that reached clinical trials. Fusion peptides were prepared comprising sequences 316–339 and 628–647 from HER-2 connected via a GPSL linker to a measles virus fusion (MVF) protein sequence and an emulsion adjuvant.^317^ The combination vaccines were observed to have good safety and efficacy in eliciting antibody responses. This study built on earlier work in which HER-2 B cell epitopes were coupled to a promiscuous T cell epitope from MVF 288–302.^318,319^ This multiepitope vaccine (along with IL-12) led to significant reduction in the quantity of pulmonary metastases resulting from challenge with tumor cells overexpressing HER-2. Leading B cell epitope candidates were identified based on computer modeling of antibody binding, from which peptides were selected for further study including the two sequences mentioned above.^319^ The crystal structure of human HER-2 complexed with trastuzumab shows that the antigen-binding region of HER-2 covers residues 563–626 and that there is extensive disulfide-bonding.^121^ Minimal peptides from this sequence that mimic the binding epitope were prepared, specifically four constructs were designed (Table 4) to contain as least one section from the three binding sequences that make contact with trastuzumab as well as one or more disulfide bonds in three cyclic peptides prepared. The 597–626 epitope, VAR**C**PSGVKPDLSYMPIWKFPDEEGA**C**QPL (bold cysteines highlight disulfide cross-link locations) linked to a MVF sequence via a GPSL spacer was particularly effective in generating rabbit antibodies that recognized HER-2. It also inhibited *in vitro* proliferation of HER-2-expressing breast cancer cells and produced antibody-dependent cytotoxicity, and immunization significantly reduced tumor burden in the mouse model studied.^121^ The same concept was used to identify cyclic peptides able to mimic the binding region of pertuzumab with the HER-2/neu dimerization domain.^320^ Again, a cyclized epitope (266–296) was linked to an MVF sequence, and the resulting construct inhibited mammary tumor growth *in vivo*. A combination of two peptides both containing the MVF sequence, one bearing the cyclized epitope 597–626 from the trastuzumab binding sequence and the other the 266–296 pertuzumab domain, has been developed (with adjuvant) and evaluated in phase II clinical trials involving patients with solid metastatic tumors (lung, breast, colon, ovarian, and others)^321^ and is known as B-Vaxx.^25,322^

**Table 4:**
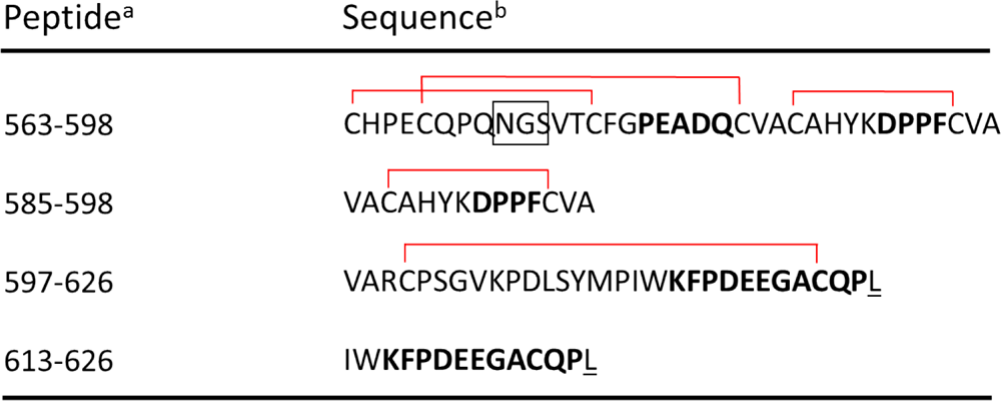
Peptides Synthesized from the Trastuzumab-Binding Domain of HER-2^121^

a Peptides containing disulfide bonds (red lines) are shown; linear versions were also synthesized.

b Residues involved in binding trastuzumab are shown in bold, and the box highlights a possible *N*-linked glycosylation site in the 563–598 epitope. Underlined amino acids show Cys to Lys mutations to avoid disulfide formation.

Peptides that could act as Th epitopes have been investigated based on HER-2 sequences 369–384 (KIFGSLAFLPESFDGDPA), 688–703 (RRLLQETELVEPLTPS), and 971–984 (ELVSEFSRMARDPQ).^115^ These sequences contain HLA-A*02-binding motifs in residues 369–377, 689–697, and 971–979. These peptides were shown to provide effective antitumor CD8^+^ T cell mediated immunity and were able to lyse tumors in breast or ovarian cancer. Th epitopes of the HER-2/neu protein mixed with GM-CSF have been developed as vaccines for HER2/neu-overexpressing ovarian, breast, or non-small-cell lung cancers.^323^ Epitopes employed include several each from extra- and intracellular domains of the protein. The final HLA-A*02 vaccine formulation consisted of peptides corresponding to the above HER-2 sequences 369–384, 688–703, and 971–984.^323^ Work on HER-2 peptide vaccines has recently been reviewed.^324^

### 4.2 MUC1

Tumor-associated glycoprotein MUC1 (mucin 1, cell surface associated) is a target for cancer immunotherapy. It is overexpressed by cancer cells, and the glycosylated proteins can concentrate growth factors near cancer cell receptors, and the extensively glycosylated proteins can also block immune cells and therapeutic drugs. Huang et al. used the Q11 peptide (section 2) in their development of a self-assembling adjuvant-free peptide system for cancer immunotherapy.^122^ They targeted the overexpression of MUC1 proteins by epithelial cancer cells. The MUC1 variable number of tandem repeats (VNTR) domain is a B cell epitope, and this 20-residue sequence (HGVTSAPDTRPAPGSTAPPA) along with glycosylated variants (at T9 or T16) were shown to form fibrils that display B cell epitopes. The conjugates glycosylated at T9 were shown to generate an immune response, that is, measurable IgG titers, in mice. The antisera were also analyzed by ELISA, and flow cytometry was used to probe binding of antibodies to MCF-7 human tumor cells that express MUC1.^122^

**Figure 15:**
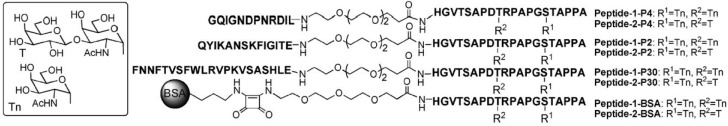
Structures of MUC1 glycopeptides **1** and **2**, conjugated with T helper epitopes **P2**, **P4**, **P30**, and BSA. Reproduced with permission from ref (76). Copyright 2013 Wiley-VCH.

A self-adjuvanting MUC1-based vaccine was designed by Cai et al.^76^ They coupled the tandem repeat glycopeptide HGVTSAPDTRPAPGSTAPPA from MUC1 to three universal Th cell epitope peptides from tetanus toxoid:^74−76^ P4(GQIGNDPNRDIL), P2 (QYIKANSKFIGITE), and P30 (FNNFTVSFWLRVPKVSASHLE) (Figure 15). These peptides can be used to substitute for the parent tetanus toxoid and can stimulate human and mouse immune systems. Raju et al. determined the sequences of tetanus toxin (TTX) recognized by CD4^+^ T cell lines stimulated with TTX or with a large pool of 20-residue synthetic peptides, overlapping by five residues and spanning the complete sequences of the TTX light (L) and heavy (H) chains.^325^ The authors noted that the peptide pool lines did not completely match the T cell reactivity of the full protein, and that this needs to be considered when peptide-propagated lines are used in T cell repertoire studies.^325^ Brossart et al. used computational sequence analysis to identify HLA-A*02 binding peptides derived from the MUC1 protein for vaccine therapies.^326^

Tecemotide is a lipopeptide antigen used in a MUC1 cancer vaccine. The vaccine contains lipopeptide STAPPAHGVTSAPDTRPAPGSTAPPKG where K denotes palmitoyl-lysine. This reached phase III trials for non-small-cell lung cancer (START).^327^

### 4.3 NY-ESO-1

NY-ESO-1 is an antigen expressed in a range of cancers (originally identified as a testicular cancer antigen),^328,329^ as well as in normal testes, and is therefore considered as a potential target for the development of vaccines against a number of epithelial cancers.^128,330^ This antigen is present in 80% of synovial cell sarcoma patients and about 25% of those with common epithelial tumors or melanoma.^331^ The NY-ESO-1 gene was identified by analysis of recombinant cDNA expression libraries using autologous patient serum antibodies and tumor mRNA.^328^ The sequence of this 180-residue peptide (miniprotein) has also been provided.^328^ Peptide NY-ESO-1 157–170 (SLLMWITQCFLPVF) contains both Th and CTL epitopes and as such is recognized by NY-ESO-1-reactive CD8^+^ and CD4^+^ T cell clones. Furthermore, it shows promise as a cancer treatment since both CD4^+^ and CD8^+^ T cells are produced in blood from melanoma patients after *in vitro* stimulation with this peptide.^128^ Autologous CD4^+^ T cells with specificity to NY-ESO-1 (specifically DCs pulsed with the SLLMWITQCFLPVF epitope co-cultured with patient T cells) have been used in the treatment of metastatic melanoma.^332^ A shorter epitope SLLMWITQC was used in clinical trials of an immunotherapy for metastatic synovial cell sarcoma or melanoma using genetically engineered lymphocytes (TCRs recognizing the peptide epitope) reactive with NY-ESO-1.^331,333^ The same sequence was also used in clinical studies with patients having myeloma.^334^ NY-ESO-1 specific TCR engineered T-cells were found to produce sustained antigen-specific antitumor effects.

CD4^+^ responses to a range of NY-ESO-1 peptides have been assessed in cancer patients.^129^ NY-ESO-1 peptide 80–109 (LLEFYLAMPFAT) was found to be the most immunogenic, although other 12-mer peptides also elicited a T-cell response.^129^ A phase I trial of a vaccine for NY-ESO-1 related cancers was performed on patients with esophageal cancer, non-small-cell lung cancer, and gastric cancer.^335^ The 20-mer peptide YLAMPFATPMEAELARRSL (NY-ESO-1, 91–110) which incorporates multiple epitopes recognized by CD4^+^ and CD8^+^ cells, as well as antibodies, was used together with adjuvant. Both CD4^+^ and CD8^+^ T cell responses and NY-ESO-1 antibodies were increased or induced in nearly all patients.^335^

Peptides derived from NY-ESO-1 have been employed in phase I clinical trials for prostate cancer vaccines.^41^ The most immunogenic peptides (restricted by HLA-A*02 and specific haplotypes) were employed, specifically DP4-restricted NY-ESO-1 peptide YGRKKRRQRRRSLLMWITQAFLPV, DR4-restricted NY-ESO-1 peptide PGVLLKEFTVSG (ESO DR4-1P), and the A2-restricted peptide SLLMWITQC (fragment NY-ESO-1 157–165).^41^

### 4.4 Folate Receptor

Folate (vitamin B_9_) is an essential compound with an important role in cell growth and division. Insufficient intake of folate may increase the risk of cancers including those of the breast, ovary, pancreas, lung, brain, cervix, and prostate gland. Folate is transported into the cell via the folate receptor, the reduced folate carrier (RFC), and the proton-coupled folate transporter (PCFT).^25^ Folate receptor-A (FR-α) overexpression is associated with many cancers such as those mentioned above.^336^

Malonis et al. have tabulated peptide sequences from FR-α (one of the two membrane-associated forms of FR) used in vaccines.^25^ This includes E39 (EIWTHSTKV, FR-α 191–199), which was used in a phase I clinical trial for advanced stage ovarian cancer in a vaccine incorporating this peptide along with four other MHC class I peptides, one MHC class II peptide, and an adjuvant.^77^ The ovarian-cancer protein-derived peptides were derived from HER-2/neu (see section 4.1) or melanoma-associated antigen-A1 (MAGE-A1), as well as FR-α and the MHC class II peptide AQYIKANSKFIGITEL derived from tetanus toxoid protein. The trials showed low toxicity but limited T cell responses.^77^

Epitopes of FR-α were identified using a CD4^+^ cell epitope prediction algorithm, and tested for the generation of immunity in ovarian or breast cancer patients compared to controls.^337^ Fourteen peptides were identified within the carboxy- and amino-termini of FR-α. It was found that a significant proportion of patients showed immunity against at least one peptide.^337^ On the basis of these results, a phase 1 clinical trial using five FR-α peptides (with GM-CSF adjuvant) was launched involving ovarian and breast cancer patients.^338^ The vaccine was reported to be well tolerated in all patients and to elicit or augment immunity in more than 90% of patients. Phase II clinical trials of this multi-antigen mixture (combined with monoclonal antibody durvalumab) are being pursued by Marker Therapeutics as a treatment for ovarian (and breast) cancer.^339^

An 12-mer peptide (MHTAPGWGYRLS) specific for FR-α was isolated from a phage library of random dodecapeptides.^127^ The tumor targeting ability of this peptide was examined via phage homing and fluorescence imaging experiments.^127^ Phage display screening of therapeutic peptides for other cancers has been reviewed.^340,341^ Tumor-associated lymphocytes recognize other peptides derived from folate binding proteins and a number of nonameric peptides with this function have been identified.^342^

### 4.5 Prostate-Specific Antigen

Prostate-specific antigen (PSA) is produced in the prostatic epithelium, in both benign and malignant forms, and its level is elevated in cancer of the prostate. PSA-based vaccines for prostate cancer have been reviewed.^343^ A clinical trial of peptide PSA 154–163 (155L, i.e., VLSNDVCAQV) progressed to phase II trials.^42^ Although a CD8^+^ T cell response to the native peptide PSA_154–163_ (VISNDVCAQV) was induced, the modified agonist peptide failed to stimulate reactivity against tumor targets expressing PSA.^42^ The modified VLSNDVCAQV sequence had been identified in earlier studies on binding of PSA epitopes to HLA and T cell activation.^123,124^ Peptide FLTPKKLQCV (PSA_154–163_) also shows HLA-A*02 binding and CTL responses.^123^

Earlier work led to phase I/II clinical trials of a T cell therapy for prostate cancer using autologous DCs exposed to peptides specific for HLA-A*02:01 from prostate-specific membrane antigen (PSMA) [an enzyme also known as glutamate carboxypeptidase II or *N*-acetyl-l-aspartyl-l-glutamate peptidase I].^344−346^ Peptides LLHETDSAV and ALFDIESKV were used, and cellular responses were detected along with a decrease in PSA level in some patients who received DCs exposed to the latter peptide, supporting potential utility in prostate cancer therapy,^344^ and a combination of the two peptides was used in phase II trials.^345−347^ The responses observed in the clinical trials were generally significant (some partial responders were noted) and of long duration.^345^

Peptides from prostate stem cell antigen (PSCA) have also been used in the development of immunotherapy for advanced prostate cancer.^126^ Human T cells could recognize the peptides in a HLA-A*02:01 specific fashion. Peptide PSCA_14–22_, ALQPGTALL, was able to generate a T cell response specific to PSCA in a human lymphocyte culture from a patient with metastatic prostate cancer.^126^ Peptides (nonamers) from prostatic acid phosphatase (PAP), a prostate tissue-specific antigen that binds HLA-A*02, were identified.^125^ The lead peptide ALDVYNGLL was used to generate tumor-specific CTLs in a study of PAP-based antigens for immunotherapy in prostate cancer.^125^

Phage display of 12-mer peptides led to the identification of PSMA peptide ligands, with lead candidate GTIQPYPFSWGY shown to bind strongly and specifically to androgen-sensitive human prostate adenocarcinoma (LNCaP) cells.^348^ This peptide is able to deliver the pro-apoptotic peptide d-(KLAKLAK)_2_ to LNCaP cells causing cell death, although studies on immunotherapy using this sequence have not as yet been performed.

### 4.6 T Cell Interactions and TCR-Based Vaccines

**Figure 16:**
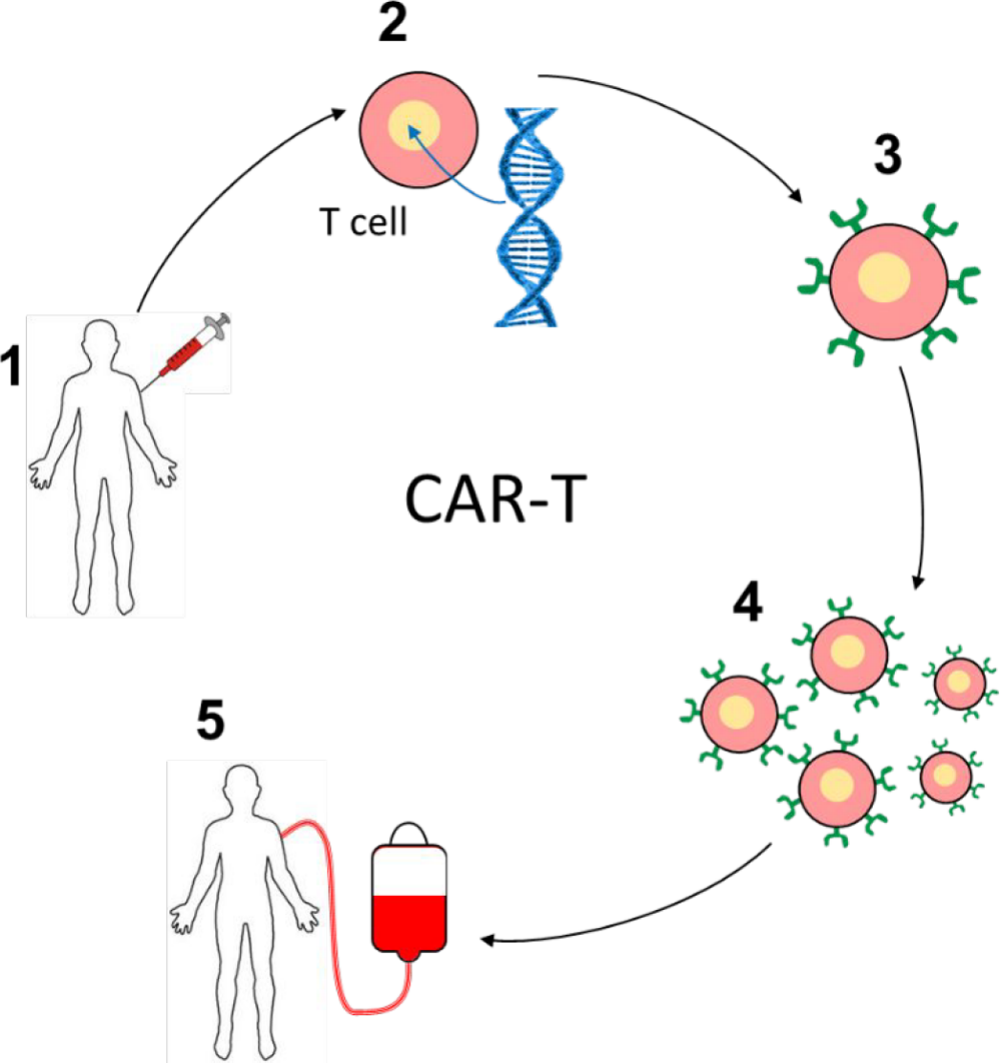
Schematic of chimeric antigen receptor T cell (CAR-T) therapy. In stage 1, T cells are extracted from blood, then in stage 2, the gene encoding specific antigen receptors is incorporated *ex vivo* into the T cells, producing (3) CAR receptors labeled on the surface of cells. (4) These cells are grown and harvested before (5) engineered T cells are infused back into the patient.

Chimeric antigen receptor T cell therapy (CAR-T) (Figure 16) is emerging as an important route for cancer treatments, especially for challenging solid tumors. It has the potential for personalized cancer vaccines, using patient-derived cells. Peptide epitopes recognized by T cells that can promote their cancer cell killing activities are thus of considerable interest. Arrays of autoimmunogenic tumor antigens have been created by identifying the antigenic targets on cultured melanoma cells recognized by CTLs.^349^ This is usually used to identify genes rather than peptides; however a method to identify antigens recognized by CTL on most HLA-A*02 melanomas involves the isolation and sequencing of the peptides displayed by class I MHC molecules at the cell surface.^350^ Peptide epitopes that lead to cancer immunogenicity have also been identified based on predicted binding to MHC class I and the conformational stability of the interacting peptide–MHC class I complex.^351^ Tumor antigens in T cell immunotherapy have been reviewed.^349^ Vaccination with peptides can enhance the activity of CAR-Ts. In one example,^352^ solid tumors have been shown to suppress tumor-specific immune responses via multiple mechanisms, and this is a significant factor in the development of adoptively transferred (patient-derived) tumor-specific T cell therapies.^352^ Since viruses induce potent immune responses, it has been proposed that their immunogenicity can be used in the treatment of solid tumors using virus-specific T cells engineered to incorporate tumor-specific chimeric antigen receptors.^352^ Tanaka et al. examined the activation of T cells specific for VZV (varicella zoster virus) using overlapping peptide libraries spanning virion proteins of VZV.^352^ Amphiphilic CAR-T ligands have been developed that, upon injection, traffic to lymph nodes and decorate the surfaces of APCs, thereby priming CAR-Ts in the native lymph node microenvironment.^353^ The amphiphilic CAR-T ligands include peptide amphiphiles with an albumin-binding phospholipid backbone, a PEG linker, and an antibody-binding peptide sequence (epidermal growth factor receptor type III deletion mutant, EGFRvIII).^353^ Lymph node targeting is important because antigens conjugated to DC-targeting antibodies reach these cells in the draining lymph nodes as noted in a study on peptide amphiphiles (or CpG nucleotide amphiphiles) as molecular vaccines with a range of antigens.^354^

Tumor infiltrating lymphocytes (TILs) may also be targeted for adoptive cellular therapy. A screening approach was used in a genome-wide approach to identify patient tumor-expressed mutated proteins, followed by synthesis and evaluation of mutated T cell epitopes as candidates based on modeled MHC binding ability for recognition by TILs.^355^ This led to the identification of mutated antigens expressed on autologous tumor cells recognized by TILs from three individuals with melanoma where tumor regression was observed following adoptive transfer of TILs. Candidate HLA-binding epitope peptides (9 and 10 residues) were thus identified.^355^ A similar strategy was developed as a cancer immunotherapy based on mutation-specific CD4^+^ T cells in an epithelial cancer patient.^356^ The cells recognized a mutated 25-residue peptide (ERBB2IP fragment) expressed by the cancer, and regression of the tumor was observed after treatment with the TILs. In another example, a melanoma differentiation glycoprotein antigen, pMel17/gp100, has been identified via recognition by CTL clones from the peripheral blood of melanoma patients, and by TILs.^130^ Multiple gp100-derived peptides corresponding to the consensus motif for binding to HLA-A*02 antigen were recognized by from melanoma patient TILs, including gp100 209–217 (ITDQVPSFV).^357−359^ A modification of this sequence IMDQVPSFV was combined with sequence YMDGTMSQV from tyrosinase (368–376) that is recognized by human CTLs in a system used to potentially treat metastatic melanoma. These peptides were formulated in an emulsion with IFA with or without IL-12, which was observed to increase peptide-specific CTL response.^130^ This peptide has been shown to increase long-term memory and antigen-specific effector CD8^+^ T cells in melanoma patients using montanide as an adjuvant in a model vaccine.^360^ This tyrosinase peptide fragment and others have been investigated as peptide vaccines for melanoma, in a study focused on the effect of GM-CSF and KLH as adjuvants.^361^ Peptides processed from melanosome proteins, tyrosinase (QCSGNFMGF and LHHAFVDSIF) or gp100 (SSPGCQPPA), have been identified in a study on T cell responses in human melanoma, along with five neoantigens (antigens generated by mutations) in tumor cells.^131^

Combinatorial peptide libraries as well as modeling methods have been used to identify ligands for tumor-reactive CTLs. This subject has been reviewed.^362^ In one example, positional scanning of sequences of a series of decapeptides was used to identify tumor-reactive CD8^+^ T cell clones specific for the melanoma cell antigen Melan-A.^363^ The same group later used this procedure to screen the cytotoxicity of a library composed of 3.1 × 10^11^ 9-mer peptides in a positional scanning format, to search for antigens recognized by a melanoma-reactive CTL. It was noted that the identified optimal peptide (AAAPKIFYA) contains five amino acids that are identical to those at the corresponding position in the native SSX-2_41–49_-derived sequence (KASEKIFYV) [SSX = sarcoma X chromosome breakpoint protein].^133^ Yeast-display libraries of HLA decamer peptides have been used in an antigen screen of “orphan” TCRs expressed on human colorectal adenocarcinoma TILs.^364^ Four TIL-derived TCRs exhibited strong selectivity towards peptides presented in a highly diverse library of HLA-A*02:01 types (the most common human MHC class I molecule). Enhancement of T cell antigens by altering HLA-A*02:01 anchor residues has been used as a strategy to improve peptide vaccines.^365^ A combinatorial peptide library was created containing 9.36 × 10^12^ different decamer peptides, starting from the wild-type pre-proinsulin 15–24 peptide (ALWGPDPAAA) and TCR binding was analyzed via peptide–MHC tetramer binding at the cell surface and surface plasmon resonance measurements.^365^

The tumor antigen protein p53 (associated with the regulation of DNA repair and cell regulation including apoptosis) has potential in cancer therapies since the overexpression and mutation of p53 make it a promising antigen target for T cell-mediated immunotherapy. Using a mouse sarcoma model, Noguchi et al. screened 24 peptide mutations of a p53 gene (Meth A) and identified a nonapeptide, KYICNSSCM, that generate CD8^+^ and CD4^+^ T cell responses.^134^ The immunization of mice with this peptide (with incomplete Freund’s adjuvant) showed increased resistance to Meth A challenge. Using a mouse model, Lauwen et al. demonstrated a CD4^+^ Th cell response against three immunodominant p53 epitopes.^366^ Th1 immunity was induced by immunization of mice with synthetic peptide vaccines comprising the identified epitopes, and it was shown that the CD4^+^ T cell repertoire specific to p53 is not limited by self-tolerance (due to the expression of wild-type p53 in somatic tissues).^366^ The three p53 Th epitopes identified differ from the murine p53 Th epitope LGFLQSGTAKSVMCT (aa 108–121) previously identified.^135^

Tumor-specific immunity mediated by CD8^+^ T cells has also been reported in a murine lung carcinoma. Two tumor-associated antigen peptides, FEQNTAQP or FEQNTAQA, were shown to induce a CTL response.^136,137^ A CTL response was observed to a mouse vaccine based on a genetically engineered hybrid comprising the model epitope OVA_254–267_, SIINFEKL (Table 1), conjugated to a naturally occurring hepatitis B core protein nanocage.^71^

Tumor-reactive CTLs associated with melanocytes and melanoma bind to peptide antigens from the Melan-A/MART-1 gene.^138^ Melan-A-specific CTLs (HLA-A*02:01-restricted) recognize mainly the Melan-A_27–35_ (AAGIGILTV) and the Melan-A_26–35_ (EAAGIGILTV) peptides. The Melan-A_27–35_ variant containing a Leu in position 1 (LAGIGILTV) induces specific T cells *in vitro* with enhanced immunological activity compared to the native peptide.^140^ An analogue of the Melan-A_26–35_ peptide with A2L substitution displayed stable binding to HLA-A*02:01 and was also better recognized than the natural peptide by tumor-reactive CTL clones.^138^ It has been shown that MART-1 tumor-specific T cells are activated after immunization with this and closely related peptides and different adjuvants.^367^ Further information on vaccines based on MART-1 and other peptide vaccines is available in a review.^302^ The AAGIGILTV peptide and the tyrosinase peptide YMDGTMSQV are susceptible to enzymatic degradation by dendritic cells, which has to be considered for *in vivo* applications (to prevent or reduce this requires sequence modifications, extensions, etc.).^139^

Another study based on Wilms’ tumor gene (this gene is overexpressed in most types of leukemia and several solid tumors, including breast and lung cancer) WT1 peptides examined binding of epitopes to particular HLA-A*02 molecules. The modified peptide (CYTWNQMNL) was more effective in eliciting CTLs specific for WT1 than the natural WT1 peptide.^141^

Peptides associated with major histocompatibility complex (MHC) class I expressed by tumors are recognized by CTLs; however many such peptides are weak immunogens and thus so-called heteroclitic peptides (synthetic variants of natural sequences) have been designed to enhance immunogenicity. This was demonstrated, for example, in a study using a peptide based on a herpes simplex virus (HSV) glycoprotein sequence B_498–505_, SEIEFARL, which also serves as a model tumor antigen of viral origin.^132^ This peptide differs from the native sequence SSIEFARL at position 2. The latter peptide contains serine substituting glutamic acid, which should reduce electrostatic repulsion with MHC class I molecules and hence improve MHC binding and thus immunogenicity. This heteroclitic peptide was successfully used with DNA to immunize against melanoma tumor challenge and elicit regression of tumors. In addition, H–K^b^-binding motifs (a haplotype, i.e., group of alleles, of a mouse MHC) from tyrosinase-related protein gp75 were identified and peptide TWHRYHLL (gp_222–229_) was found to have similar binding properties to SEIEFARL and was used to produce a heteroclitic variant TAYRYHLL, which was found show strong binding to K^b^, comparable to SSIEFARL. This heteroclitic peptide, based on a self-antigen expressed by melanoma cells, was also used (with DNA) in a vaccine that conferred protection against tumors in mice.^132^

### 4.7 Peptide Vaccines for Virus-Induced Tumors

In early work, Melief’s group identified a CTL peptide epitope able to prevent human papillomavirus (HPV)-induced tumors.^142^ HPV infection is responsible for 90% of cervical cancer cases, the commonest being the HPV-16 subtype, and two genes, E6 and E7, play major roles in the progression of the malignant phenotype.^368^ A series of 240 overlapping peptides from HPV-16 E6 and E7 were evaluated in terms of their binding to H-2K^b^ and H-2D^b^ MHC class I molecules.^142,369^ This led to the identification of the H-2D^b^-binding CTL epitope E7 49–57 (RAHYNIVTF, which is part of a longer sequence identified as immunogenic *in vivo* by Tindle et al.^143^), as well as HPV E6 sequences that bind H-2K^b^.^142^ However, this group later noted that synthetic peptide immunization can also lead to CTL tolerance instead of immunity and enhanced tumor growth.^370,371^ It was also reported that short peptide epitopes such as OVA_257–264_ do not permanently stimulate CD8^+^ T cells (this is contradicted by work from Collier’s group discussed in section 2) although they found that longer sequences can do so.^372^ Synthetic peptides comprising the sequence DRAHYNI (E7_48–54_) conjugated to major B cell epitopes on the E7 molecule can elicit strong antibody responses to HPV-16 E7.^143^ This is a good example of a peptide vaccine in which linked Th and CTL epitopes provide a strong immune response in mice.^373^ Vaccines that combine E7 subunits with conventional therapeutics such as cisplatin can offer enhanced chemotherapeutic activity, for example, through increased susceptibility to the killing of cisplatin-treated tumors mediated by CTLs.^374^

In a study on lipopeptides containing a peptide sequence, STDSCDSGPSNTPPEI, from human adenovirus type 5 early region 1B (Ad5E1B), Melief and coworkers have reported that CTL tolerance is also not suppressed by peptide lipidation or incorporation into liposomes (in fact these cause tumor outgrowth), although it can be avoided by presentation of peptides on dendritic cells.^78^ An *in vivo* study again using OVA peptide fragments showed that extended peptides are presented selectively by activated DCs whereas short peptides are also displayed by T cells and B cells.^375^ Experiments using B cell knockout mice revealed that B cells have an important role in the priming of T cells for short peptides but not for long peptides.^375^ The issue of tolerance, especially in relation to cross-presentation of cellular antigens, has also been reviewed.^376^ A low binding affinity of minimal peptides, often derived from “self” sequences (involved in the established T cell response), to the MHC can be insufficient to activate CTL cells. To increase the immunogenicity of peptide vaccines, the MHC–peptide complex can be stabilized. In one example, this has been achieved by modification of cysteine residues.^377^ It was shown that ensuring that these are present in reduced form leads to a 10–100-fold increase in antigenicity of two influenza virus nucleoprotein (NP) peptides, although this is not related to the affinity to H-2K^d^. Similar enhancements were obtained by substituting cysteine with alanine or serine in the synthetic peptides.^377^ That immunogenicity requires high affinity MHC class 1–peptide binding was confirmed by a study using 83 peptide epitopes, which also established that CTL binding capability is also necessary.^378^ In particular, high affinity H–K_b_-binding peptides induced peptide-specific CTL responses. In a study based on HBV and HPV-16 peptide epitopes, it was also confirmed that those that form stable MHC–peptide complexes exhibit immunogenicity.^379^ Many HLA-A*0201-restricted T cell epitopes unfortunately form low dissociation constant complexes.^379^

In a study of T cell activation parameters to predict vaccine efficacy using a range of TLR agonists, the HPV-16 E7 epitope RAHYNIVTF has been used as well as the HPV-16 E7 35-residue long peptide QAEPD**RAHYNIVTF**CCKCDSTLRLCVQSTHVDIR (aa 43–77) spanning both the Th epitope (underlined) and the CTL epitope (in bold), along with a 32-mer peptide LPDEVSGLEQLESIINFEKLTEWTSSNVMEER that encodes the OVA epitope SIINFEKL discussed in section 2.^69^ A series of adjuvants comprising TLR agonists and an agonistic CD40-specific antibody activated DCs *in vitro* although a strong functional T cell response *in vivo* was not induced in all cases.

In a preclinical study of cervical cancer induced by HPV-16, immunization with a HPV-16-derived 35 amino acid extended peptide that contains both CTL and Th epitopes was tested by this group compared to immunization with a minimal HPV-16 E7 9-residue peptide CTL epitope.^373,380^ The HPV-16 E7 long peptide vaccine induced pronounced HPV-16-specific CD4^+^ and CD8^+^ T cell immunity in mice. In mice vaccinated with the 35-residue peptide vaccine, but not the minimal CTL peptide, HPV-16-positive tumors were eliminated.^380^ Based on these studies, a phase I/II clinical trial of a vaccine consisting of a series of 13 overlapping peptides was launched in end-stage cervical cancer patients, each peptide comprising 27–35 residues spanning the complete sequence of the HPV-16 E6 and E7 proteins.^381^ Vaccine-induced T cell responses specific for HPV-16 E6 were detected in all patients in the study, and HPV-16 E7-specific T cell responses were observed in five out of six patients.^382^ Despite this, and good tolerance, only limited therapeutic activity was noted.^373^ The same group also carried out a phase II vaccination study to treat HPV-16-positive vulvar intraepithelial neoplasia grade III. A vaccine containing the 13 overlapping long peptides of the E6 and E7 oncoproteins of HPV-16 was used for immunization, with some promising outcomes in terms of patients showing regression of tumors.^373^

Polyoma virus is a small DNA tumor virus, and peptides derived from the sequences of distinct size T antigens can be used to immunize against polyoma tumors in a study based on a mouse model.^144^ Peptides derived from sequences found in all three T antigens (aa 1–19, MDRVLSRADKERLLELLKL) among others were all shown to induce immunity against polyoma tumors.^144^

Tumors caused by murine leukemia virus (MuLV) can be prevented by vaccination with Th cell epitopes (EPLTSLTPRCNTAWNRLKL and SSWDFITV) from the virus.^145^ The peptide-specific CD4^+^ T cells generated did not directly recognize tumor cells; thus tumor-associated APCs may be cross-primed. CD8^+^ CTLs that recognize an immune-dominant viral gag-encoded CTL epitope were the main effector cells in the elimination of tumors.^145^ In a related study, it was observed that immunization with a CTL epitope, SPSYVYHQF, from the tumor cell-expressed MuLV gp70 envelope protein, does not protect BALB/c mice against challenge with CT26 tumor cells.^383^ However, combining this peptide with a Th peptide, OVA 323–337 or sperm whale myoglobin (SWM) 106–118, elicited Th cell responses and protected fractions of the mice immunized.^383^ The conclusion that both CD8^+^ and CD4^+^ T lymphocytes are required for immunity is also supported by a study showing that tumor-associated lymphocytes can be isolated from BALB/c mice injected with tumor cells using SV40 large T antigens.^384^

## 5 Concluding Remarks

As exemplified by the many remarkable studies discussed in this review, peptides can have considerable potential in the design of antigens or adjuvants in vaccines. Inevitably, there are both advantages and disadvantages to the use of peptides. A major advantage, exemplified by many examples in this review, is the ability to produce highly selective and specific antigens, based on natural immunogenic epitopes. Also, peptides can be selected or designed in order to ensure a good safety profile, avoiding undesirable immune responses or other side effects. In addition, peptides are easy to design and synthesize (with the potential also to scale-up using established techniques), and it is possible to control peptide conformation and (if it occurs or is desired) self-assembly using established physicochemical principles. Other advantages include the availability of methods to prepare high purity peptides, with reduced biological impurities, thus reducing potential allergic reactions. Another advantage of peptides is that their immunogenicity can be subjected to preliminary assessment using *in silico* methods, potentially followed by *in vitro* techniques, to examine binding of peptides in MHC complexes or to HLAs. This is becoming increasingly widespread, as shown, for example, by the many examples of *in silico* modeling of SARS-CoV-2 interactions with cells and immunogenicity and toxicity in 2020 and 2021 following the global COVID-19 outbreak, discussed further in section 3.5. Despite the increased use of many different web servers to predict peptide immunogen properties, it remains a significant challenge to predict the actual *in vivo* immune response to a given antigen. The translation to practice still requires extensive trial-and-error studies and animal trials before testing on humans. It should be emphasized that the immune responses of animals can be very different from those of humans, due for instance to significant differences in the PRRs displayed in human cells compared to those of animals.

Disadvantages of peptide vaccines include issues of biostability, that is, the susceptibility to proteolysis of peptides containing native l-amino acid residues; for example this has been shown for MHC-bound peptides in studies examining expression by hybridomas^67,385^ as well as DC surface proteases.^139^ The limited stability of peptides *in vivo* can be avoided by using non-natural amino acids, cyclization, or peptidomimetics among other approaches.^13,22^ It has also been emphasized that longer peptides may be used to reduce T cell tolerance and extend the time scale of *in vivo* epitope presentation by professional APCs.^373,375^ The constrained conformations of short peptides may also be problematic; since in general they will not have the three-dimensional folded structure of a protein or longer peptide, this will potentially reduce binding to human cells compared to full antigens such as those from virus coat proteins. Longer epitopes may also lead to enhanced presentation and induction of T cell expansion *in vivo* when the Tc epitope concerned displays weaker MHC class I binding.^375^ Since many peptide epitopes show limited immunogenicity (compared to vaccines prepared from attenuated viruses, for example),^25^ their practical application may involve formulation with adjuvants to boost the immune response. More broadly, the delivery of peptide subunit vaccines has to be considered. This may require preparation as nanoparticles (for example, virus-like particles), or in an emulsion or as mentioned in one example, by incorporation of cell-penetrating peptides or cell-targeting nucleic acids in the formulation. The small size of peptides can cause renal filtration, so conjugation to lipid chains,^12,44,45^ PEG chains,^386−388^ albumins,^389−392^ etc. can be used to improve half-life in circulation. Peptide-based therapies with an extended-release profile may also be useful for therapies for chronic conditions, reducing the need for daily drug administration. Slow release systems can be produced through suitable formulation in emulsion or hydrogel depots, for example. The pharmacokinetics and pharmacodynamics of a peptide therapeutic such as a subunit vaccine are both important considerations for practical purposes, and this will of necessity be examined during advanced clinical trials.

Cancer immunotherapy is a potential type of personalized medicine, that is, a personalized cancer vaccine (PCV),^297^ when for a given patient tumor sequencing and tumor-associated antigen analysis and preparation are used to select peptides for a “personalized” formulation. This has great potential for future therapeutics. The investigation of combination therapies, using peptide immunogens along with conventional anticancer drugs, is another promising area of future research (this comment also applies for treatments for infectious diseases).

As highlighted in this review, there is great potential to apply peptide epitope vaccines as therapeutics to treat infectious diseases, which affect many people, and also in tumor immunotherapy, to the possible benefit of millions suffering from cancer. This is exemplified through the many clinical trials in progress as well as intense research activity in this field.
